# Single dose recombinant VSV based vaccine elicits robust and durable neutralizing antibody against Hantaan virus

**DOI:** 10.1038/s41541-024-00814-2

**Published:** 2024-02-10

**Authors:** Hui Zhang, He Liu, Jing Wei, Yamei Dang, Yuan Wang, Qiqi Yang, Liang Zhang, Chuantao Ye, Bin Wang, Xiaolei Jin, Linfeng Cheng, Hongwei Ma, Yangchao Dong, Yinghui Li, Yinlan Bai, Xin Lv, Yingfeng Lei, Zhikai Xu, Wei Ye, Fanglin Zhang

**Affiliations:** 1 Department of Microbiology, School of Preclinical Medicine, Airforce Medical University: Fourth Military Medical University, Xi’ an, Shaanxi China; 2 Center for Disease Control and Prevention of Shaanxi Province, Xi’an, Shaanxi China; 3 Center of Clinical Aerospace Medicine, Airforce Medical University: Fourth Military Medical University, Xi’ an, Shaanxi China; 4 Student Brigade, School of Preclinical Medicine, Airforce Medical University: Fourth Military Medical University, Xi’ an, Shaanxi China

**Keywords:** Live attenuated vaccines, Live attenuated vaccines

## Abstract

Hantaan virus (HTNV) is a pathogenic orthohantavirus prevalent in East Asia that is known to cause hemorrhagic fever with severe renal syndrome (HFRS), which has a high fatality rate. However, a Food and Drug Administration (FDA)-approved vaccine is not currently available against this virus. Although inactivated vaccines have been certified and used in endemic regions for decades, the neutralizing antibody (NAb) titer induced by inactivated vaccines is low and the immunization schedule is complicated, requiring at least three injections spanning approximately 6 months to 1 year. Replication-competent vesicular stomatitis virus (VSV)-based vaccines provide prolonged protection after a single injection. In this study, we successfully engineered the HTNV glycoprotein (GP) in the VSV genome by replacing the VSV-G open reading frame. The resulting recombinant (r) rVSV-HTNV-GP was rescued, and the immunogenicity of GP was similar to that of HTNV. BALB/c mice immunized with rVSV-HTNV-GP showed a high titer of NAb against HTNV after a single injection. Notably, the cross-reactive NAb response induced by rVSV-HTNV-GP against Seoul virus (an orthohantavirus) was higher than that induced by three sequential injections of inactivated vaccines. Upon challenge with HTNV, rVSV-HTNV-GP-immunized mice showed a profoundly reduced viral burden in multiple tissues, and inflammation in the lungs and liver was nearly undetectable. Moreover, a single injection of rVSV-HTNV-GP established a prolonged immunological memory status as the NAbs were sustained for over 1 year and provided long-term protection against HTNV infection. The findings of our study can support further development of an rVSV-HTNV-GP-based HTNV vaccine with a simplified immunization schedule.

## Introduction

Rodent-borne *Orthohantavirus* (hereafter referred to as hantavirus) infection can cause two severe clinical diseases, namely, hantavirus pulmonary syndrome (HPS) caused by the New World Hantavirus (NWH) and hemorrhagic fever with severe renal syndrome (HFRS) caused by the Old World Hantavirus (OWH), which have fatality rates greater than 35% and up to 5–15%, respectively^1^. The causative agents of HFRS are Hantaan virus (HTNV), Seoul virus (SEOV), Dobrava-Belgrade virus (DOBV), and Puumala virus (PUUV), which are found mainly in Europe and Asia. The clinical manifestations of HFRS caused by different hantavirus infections vary from subclinical, mild, and moderate to severe; among them, HTNV and DOBV are responsible for more severe symptoms^2^. Annually, over 85% of the approximately 50,000 cases worldwide occur in China alone, and they are mainly caused by HTNV and SEOV and pose a significant threat to human health, especially in rural areas^3^. At present, cell culture-based HTNV and SEOV bivalent inactivated vaccines are used in areas endemic for the disease in China^4^. Although it is effective in inducing an antibody response after inoculation, the duration of the neutralizing antibodies (NAbs) is extremely short. Thus, developing a safe and effective prophylactic vaccine with a high titer and durable NAb response is urgently required to control hantavirus infections.

Belongs to the order *Bunyavirales*, family *Hantaviridae*, and genus *Orthohantavirus*, HTNV is an enveloped, trisegmented, negative-sense ribonucleic acid (RNA) virus. The small (S) segment encodes the nucleocapsid protein, the medium (M) segment encodes the glycoprotein precursor (GPC), and the large (L) segment encodes the RNA-dependent RNA polymerase (RdRp)^5^. GPC is cleaved by host proteases at the conserved WAASA pentapeptide site into two mature envelope glycoproteins, glycoprotein N (Gn) and glycoprotein C (Gc)^6^. Both Gn and Gc are type I transmembrane proteins and the sole glycoproteins on the virion surface. Thus, both Gn and Gc serve as protective antigens against NAbs in vivo. Other Gn/Gc-based vaccines have also been developed, such as virus-like particle (VLP)-based, deoxyribonucleic acid (DNA), and virus-vectored vaccines^7–13^. However, the immunization schedule for VLP and DNA vaccines often requires three sequential injections and extends for approximately 6 months to 1 year, thus posing a great challenge for improving vaccination regime adherence, especially in rural areas. For vectored vaccines, pre-existing antibodies against the vectors often hinder vaccine efficacy. Generation of a vectored vaccine without pre-existing antibodies and with a single inoculation could serve as a potential solution, which will help to elevate the immunization rate and reduce the disease burden.

Vesicular stomatitis virus (VSV) is a non-segmented, negative-sense, single-stranded RNA virus that belongs to the family *Rhabdoviridae* and genus *Vesiculovirus*. The genome contains five sequential open reading frames (ORFs) that encode nucleocapsid protein (N), phosphoprotein (P), matrix protein (M), glycoprotein (G), and RdRp (L). VSV is an animal virus; its attenuated ability to infect humans and its ability to pseudotype different viral glycoproteins have made VSV a widely accepted choice of vector for vectored vaccines since the reverse genetics strategy was established^14,15^. As replication-competent vectors, recombinant VSV (rVSV)-based vaccine candidates have been shown to strongly elicit NAbs and have been suggested to protect against multiple lethal pathogens, such as Ebola virus (EBOV), Marburg virus, Lassa virus, and Nipah virus^16–19^. An rVSV expressing the Zaire Ebola virus glycoprotein (rVSV-ZEBOV) was generally well tolerated in vaccine recipients during clinical trials. The U.S. FDA and the European Medicines Agency licensed it for use in 2019 due to its high protection and effectiveness^20^.

In this study, we constructed an infectious complementary DNA (cDNA) clone of the VSV and replaced the VSV-G segment with HTNV glycoprotein (GP), resulting in the generation of rVSV-HTNV-GP. We then characterized the immunological features of rVSV-HTNV-GP and evaluated its protection effects in a mouse model. We found that a single dose of rVSV-HTNV-GP elicited long-lasting NAbs and afforded protection against HTNV challenge. Our study paves the way for further development of a VSV-vectored HTNV vaccine and calls for future research.

## Results

### Generation and characterization of replication-competent rVSV expressing HTNV GP

**Fig. 1:**
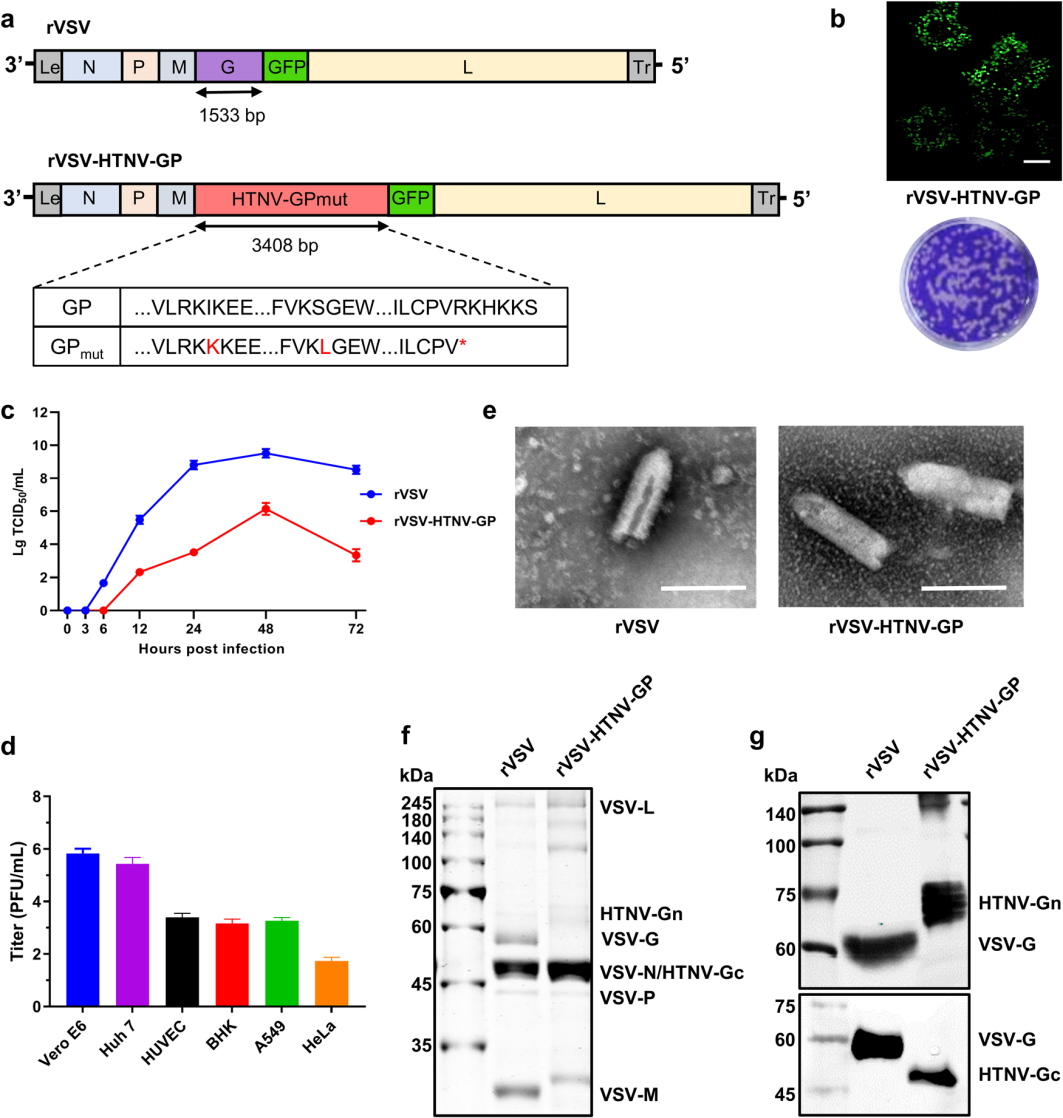
Characterization of the Replication-Competent, Infectious rVSV Chimera bearing HTNV GP. **a** Schematic diagram depicting the genomic organization of rVSV and rVSV-HTNV-GP. Shown 3´ to 5´ are the leader region (Le), nucleocapsid (N), phosphoprotein (P), matrix (M), glycoprotein (G) or HTNV GP, green fluorescent protein (GFP), large polymerase (L), and trailer region (Tr). Shown at the bottom is a schematic representation of the HTNV GP. Locations of the point mutations (I532K and S1094L) and the truncated (short by six amino acids) cytoplasmic tail of rVSV-HTNV-GP. Mutations deviating from the wild-type spike are indicated in red, and an asterisk indicates mutation to a stop codon. **b** Upper panel: Infection of Vero E6 cells with the supernatant from the cells transfected with rVSV-HTNV-GP and accessory plasmids. Images were acquired at 72 hpi using a fluorescence microscope. (Bar = 200 μm). Lower panel, plaque formed by rVSV-HTNV-GP; images acquired at 72 hpi. **c** Growth Kinetics of rVSV and rVSV-HTNV-GP strains. Vero E6 cells were infected with each virus at a MOI of 0.0001 for 72 h. TCID_50_/mL of each virus is indicated at each time point. **d** The indicated cell types were infected with rVSV-HTNV-GP at an MOI of 0.5. The supernatants were harvested at 24 hpi and titrated against Vero E6 cells using a plaque assay. **e** Electron microscopy images of negatively stained purified rVSV and rVSV-HTNV-GP particles; scale bars indicate 200 nm. **f** SDS-PAGE analysis of purified rVSV and rVSV-HTNV-GP particles. **g** Western blot analysis of purified rVSV and rVSV-HTNV-GP. VSV-G and HTNV Gn and Gc detection using the mAb VSV-G tag, Gn-1, and Gc-10.

The infectious cDNA clone of VSV with replacement in the VSV-G region by a codon optimized HTNV GP (Fig. 1a) and green fluorescent protein (GFP) marker was recovered. It expressed GFP reporter (Fig. 1b, upper panel) and formed typical plaques on Vero E6 cells (Fig. 1b, lower panel). The viral growth kinetics of rVSV-HTNV-GP were compared to those of the parental rVSV, which also contains a GFP ORF between VSV-G and VSV-L ORFs, resulting in an attenuated phenotype, with the peak titer for rVSV nearly hundred-fold that of rVSV-HTNV-GP (Fig. 1c). Negative-stain electron microscopy of iodixanol-gradient purified virus particles revealed that rVSV-HTNV-GP shared similar particle sizes and shapes with the parental rVSV particles (Fig. 1d). On subsequent testing of cell tropisms of rVSV-HTNV-GP on different cell lines. Vero E6 and human hepatoma (Huh7) cells had the highest titer, followed by human umbilical vein endothelial cells (HUVEC), baby hamster kidney (BHK)-21, and A549 cells, while Henrietta Lacks (HeLa) cells had the lowest titer (Fig. 1e); this is consistent with authentic HTNV tropisms. Purified rVSV and rVSV-HTNV-GP on subjection to sodium dodecyl sulfate–polyacrylamide gel electrophoresis (SDS-PAGE) and Coomassie brilliant blue staining, the VSV-G band was not visible in the lanes of rVSV-HTNV-GP (Fig. 1f). Furthermore, HTNV Gn and Gc expression was confirmed after western blot analysis of the virions (Fig. 1g). These results confirm that rVSV-HTNV-GP-bearing HTNV GP, instead of VSV-G, was successfully rescued.

### rVSV-HTNV-GP share surface antigenic similarity with authentic HTNV

**Fig. 2:**
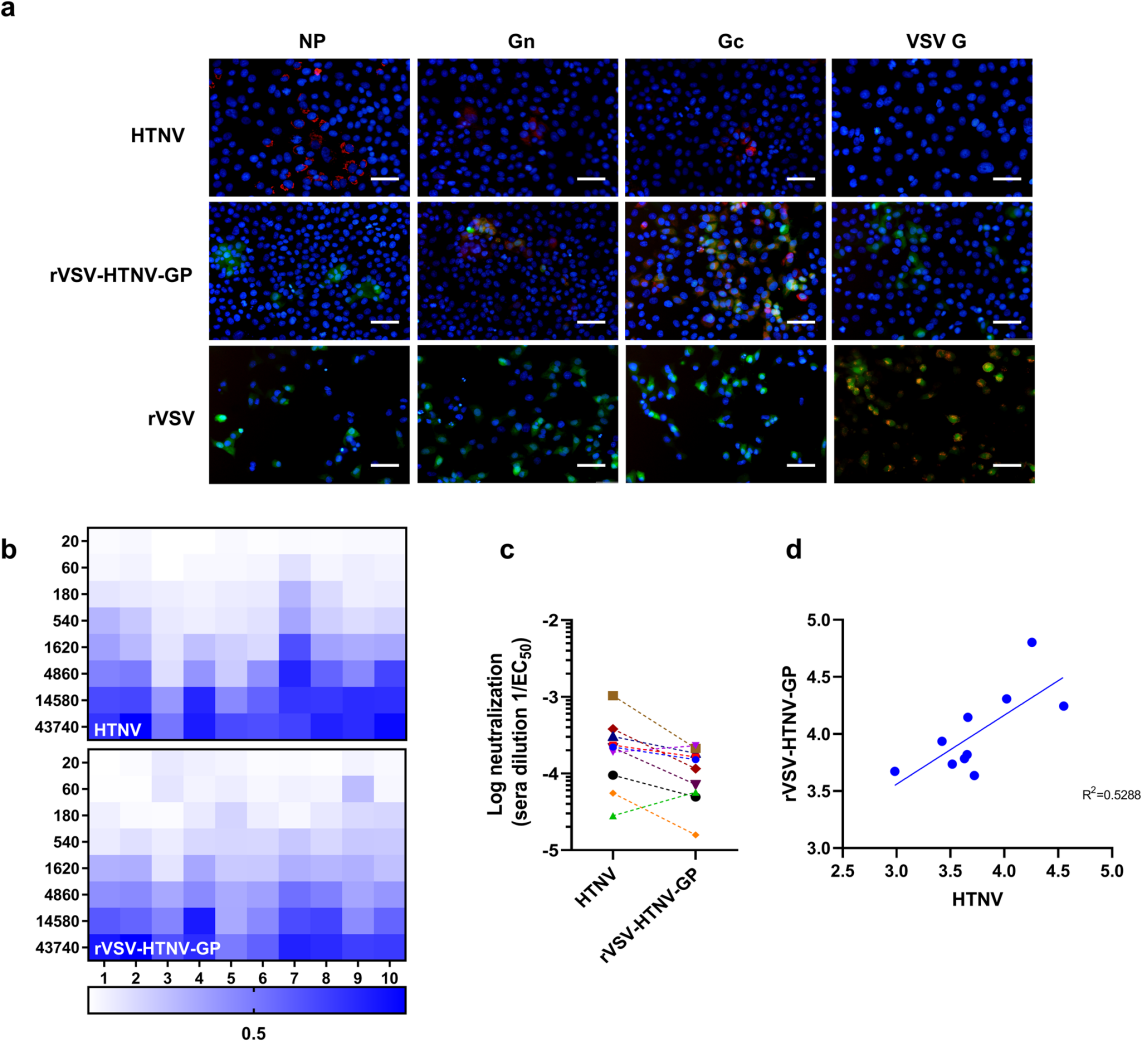
Antigenic immunogenicity between HTNV and rVSV-HTNV-GP. **a** Vero E6 cells infected with either HTNV (upper panel), rVSV-HTNV-GP (middle panel), rVSV (lower panel), and stained with antibody against HTNV NP (first column), Gn (second column), Gc (third column), and VSV G (fourth column). Scale bars: 500 µm. **b** Evaluation of neutralizing activity of HFRS convalescent sera against HTNV or rVSV-HTNV-GP. For neutralization assay of HTNV, 100 FFUs of HTNV were incubated with three-fold serial dilutions of antisera from patients with convalescent HFRS or negative control at 37 °C for 1 h, virus:serum mixtures were then applied to monolayers of Vero E6 cells. At 5 dpi, Vero E6 cells were fixed, permeabilized and the virus positive foci were visualized using mAb antibody against HTNV NP. For the neutralization assay of rVSV-HTNV-GP, 100 PFUs of rVSV-HTNV-GP were incubated with three-fold serial dilutions of sera at 37 °C for 1 h. Virus:serum mixtures were then applied to monolayers of Vero E6 cells. At 7 hpi, the cells were fixed, and the infected cells were scored based on GFP expression. Heatmaps showing the percent neutralization of authentic HTNV or rVSV-HTNV-GP by a panel of 10 antisera. Related to Supplementary Fig. S1. **c** Comparison of the neutralizing activities of antisera (log reciprocal EC_50_ values) against authentic HTNV and rVSV-HTNV-GP. **d** Linear regression analysis of EC_50_ values from panel **c**.

To evaluate antigenic similarities between rVSV-HTNV-GP and HTNV, we stained HTNV-, rVSV-HTNV-GP-, or rVSV-infected Vero E6 cells with antibodies against HTNV-nucleocapsid protein (NP),-Gn/-Gc, or VSV-G, respectively. rVSV caused significant cytopathic effects in Vero E6 cells, and the remaining cells were positive for VSV-G, but negative for HTNV-NP, Gn, or Gc (Fig. 2a). In contrast, rVSV-HTNV-GP-infected cells were positive for HTNV Gn or Gc but negative for HTNV-NP, and Gn or Gc was only expressed in GFP-positive cells, indicating that the HTNV GP ORF was successfully expressed upon viral infection (Fig. 2a). Subsequently, 10 serum samples from the HFRS convalescent plasma were selected to determine their neutralization titer against HTNV and rVSV-HTNV-GP; the trends of the curves between the groups were very similar (Supplementary Fig. 1). Heatmaps of viral infectivity revealed similar dose-dependent neutralization patterns for rVSV-HTNV-GP and authentic HTNV from each antiserum donor (Fig. 2b). However, the serum dilutions at half-maximal neutralization derived from logistic curve fits (neutralization 50% or half maximal inhibition concentration [IC_50_]) revealed a 2- to 10-fold shift toward reduced neutralization with rVSV-HTNV-GP compared with authentic HTNV (Fig. 2c). This discrepancy may be due to assay-specific differences between rVSV-HTNV-GP (half-GFP puncta formation reduction neutralization test, GRNT_50_) and authentic HTNV (half-focus reduction neutralization test, FRNT_50_) microneutralization assays. Nonetheless, the relative potencies of the antisera against rVSV-HTNV-GP and authentic HTNV were correlated (R^2^ = 0.5288) (Fig. 2d). These results suggest that the surface antigenicities of rVSV-HTNV-GP and authentic HTNV were similar. Consequently, rVSV-HTNV-GP was demonstrated to be an authentic surrogate for HTNV and was thus suitable for eliciting the desired immune response.

### Potent anti-HTNV humoral immune responses induced by rVSV-HTNV-GP vaccine candidates

**Fig. 3:**
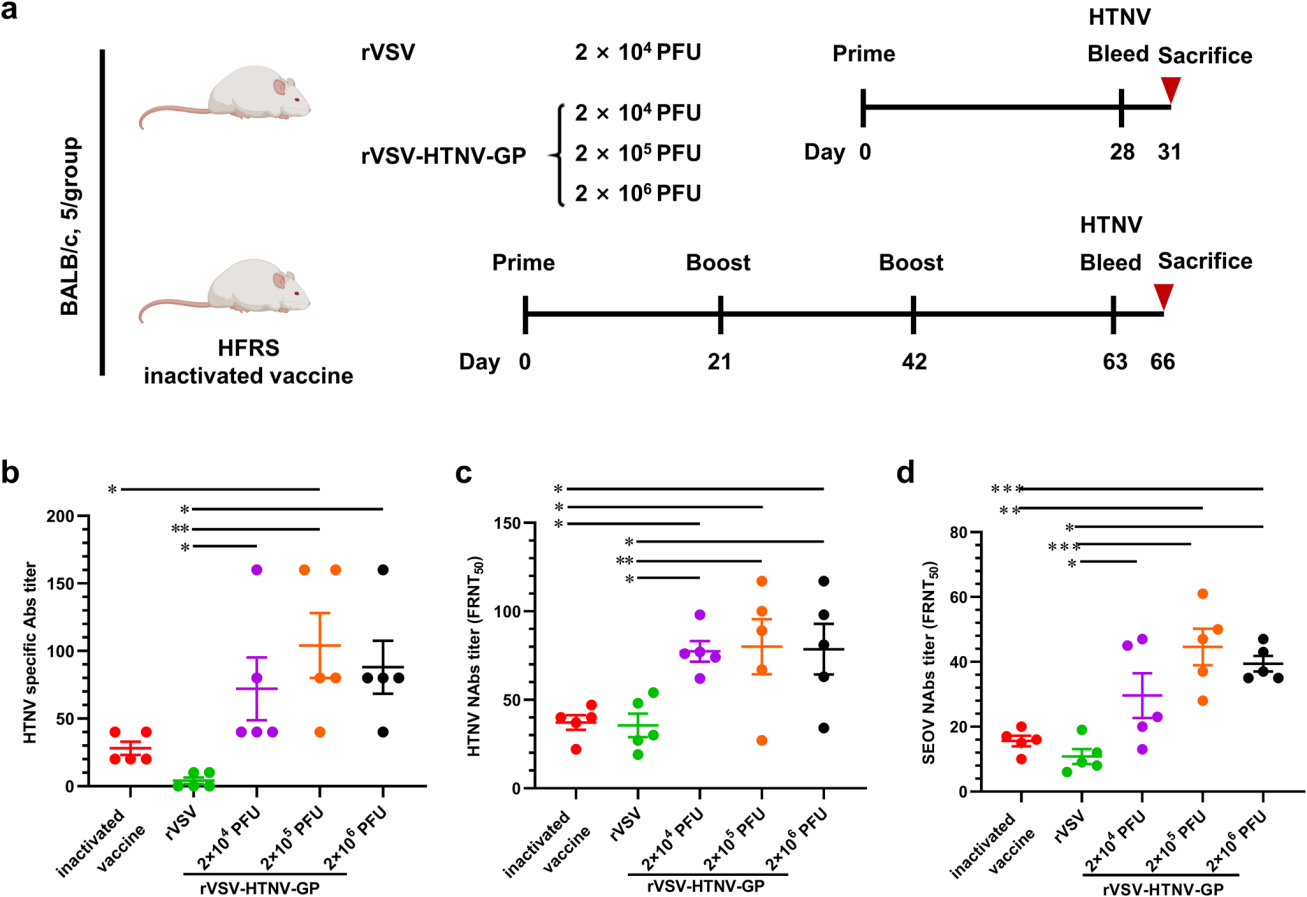
Immunogenicity of rVSV-HTNV-GP. **a** Scheme for vaccination and HTNV challenge. Six-week-old female BALB/c mice were immunized with rVSV, rVSV-HTNV-GP, or HFRS-inactivated vaccine via intraperitoneal injection (i.p.). Inactivated vaccine-immunized mice were boosted twice at 3 weeks interval after the primary vaccination. **b** HTNV specific IgG antibody responses in the sera of rVSV or rVSV-HTNV-GP vaccinated mice were evaluated 4 weeks after immunization through indirect IFA. For inactivated vaccine group, serum samples were collected 3 weeks after last boost. **c** Neutralizing antibody titers against HTNV determined by focus reduction neutralization test (FRNT). **d** Neutralizing antibody titers against SEOV, determined by FRNT. (For **b**–**d**, *n* = 5 mice per group; data are represented as the means ± Standard Errors of Mean (SEMs), one-way ANOVA with Dunnett’s post-test: *P < 0.05, **P < 0.01, ***P < 0.001).

Given that rVSV-HTNV-GP is a replication-competent vector, whether the vaccine candidates could induce specific immune responses against HTNV in a single-dose regime was examined. Six-week-old BALB/c mice were divided into four groups and immunized with three different doses of rVSV-HTNV-GP or control rVSV (Fig. 3a). An inactivated vaccine (licensed for HFRS prevention in China) inoculated with three sequential injections at 3-week intervals served as the positive control (Fig. 3a). Serum was isolated from the mice at 4 weeks post-priming with rVSV and rVSV-HTNV-GP or at 3 weeks post-final boost of the inactivated vaccine, and IgG titers against HTNV were determined, and were nearly undetectable in rVSV control group (Fig. 3b). All three rVSV-HTNV-GP groups showed significantly higher levels of IgG (mean titers of 72.0, 104.0, and 88.0 in the 2 × 10^4^, 2 × 10^5^, and 2 × 10^6^ plaque-forming units (PFUs) groups, respectively) compared with the inactivated vaccine group (mean titers of 28.0) (Fig. 3b). Although the levels of HTNV-specific IgG among the three rVSV-HTNV-GP groups were not significantly different, 2 × 10^5^ PFUs resulted in the best performance.

NAb titers against HTNV were measured using the focus-reduction neutralization test (FRNT)^21^. Immunization with 2 × 10^4^, 2 × 10^5^, and 2 × 10^6^ PFUs of rVSV-HTNV-GP-induced NAbs (mean titers of 77.4, 80, and 78.6, respectively) was two-fold higher compared to that of the inactivated vaccine (mean titer of 37.2) (Fig. 3c). Moreover, all three doses of rVSV-HTNV-GP elicited higher SEOV NAbs (mean titers of 29.6, 44.6, and 39.4) than those of the inactivated vaccine (mean titer of 13.6) (Fig. 3c). These findings demonstrate the suitability of rVSV-HTNV-GP as an immunogenic vaccine platform that elicits high titers of HTNV-specific antibodies and NAbs and induces cross-OWH NAbs against SEOV. Collectively, these data suggest that rVSV-HTNV-GP is immunogenic and elicits high titers of antibodies that neutralize HTNV GP.

In addition to 2 × 10^4^ PFUs, we also used 2 × 10^5^ and 2 × 10^6^ PFUs in the rVSV group and tested the specific antibodies and NAbs against HTNV. The experimental results showed that the specific antibodies (mean titers of 2 × 10^4^, 2 × 10^5^, and 2 × 10^6^ PFUs in the rVSV groups were 4, 2, and 2, respectively) and NAbs (mean titers of 2 × 10^4^, 2 × 10^5^, and 2 × 10^6^ PFUs in the rVSV groups were 35.6, 33.6, and 37.6, respectively) induced by the three rVSV groups were similar (data not shown). Therefore, the 2 × 10^4^ PFU group of rVSV was selected as the control in the following experiment.

### rVSV-HTNV-GP vaccine candidates induced protection against HTNV infection mainly attributed to humoral immunity

Effective vaccination involves the induction of CD4^+^ T helper (Th) cells that produce cytokines that shape subsequent humoral adaptive immune responses. To evaluate the rVSV-HTNV-GP vaccination-induced T cell responses, splenocytes collected from control and immunized mice were cultured with the HTNV GP overlapping peptide pool for examination of Th1 cytokines (interferon gamma [IFN-γ] and tumor necrosis factor alpha [TNFα]) and Th2 cytokines (interleukin [IL] IL-4) production within the cells (Supplementary Fig. 2). We observed low or no levels of Th1 and Th2 cytokines in the splenocytes of rVSV immunized mice, whereas variably high levels of Th cytokines were detected in mice immunized with rVSV-HTNV-GP vaccine candidates or the inactivated vaccine (Supplementary Fig. 3a, b), although the differences between the versatile vaccine groups were not significant. To further study vaccine-mediated cellular immunity, splenocytes collected from control and immunized mice were further examined of cytokines IL-2, IL-4, IL-10 and IFNγ production within the cells by cultured with the HTNV GP overlapping peptide pool. We observed low or no levels of cytokines IL-2, IL-4, IL-10 and IFNγ in the splenocytes of rVSV immunized mice, whereas variably high levels of Th cytokines were detected in mice immunized with rVSV-HTNV-GP vaccine candidates or the inactivated vaccine, although the differences between the versatile vaccine groups were not significant (Supplementary Fig. 4). Collectively, these results demonstrate that rVSV-HTNV-GP-induced T cell responses did not have an obvious advantage over inactivated vaccines.

**Fig. 4:**
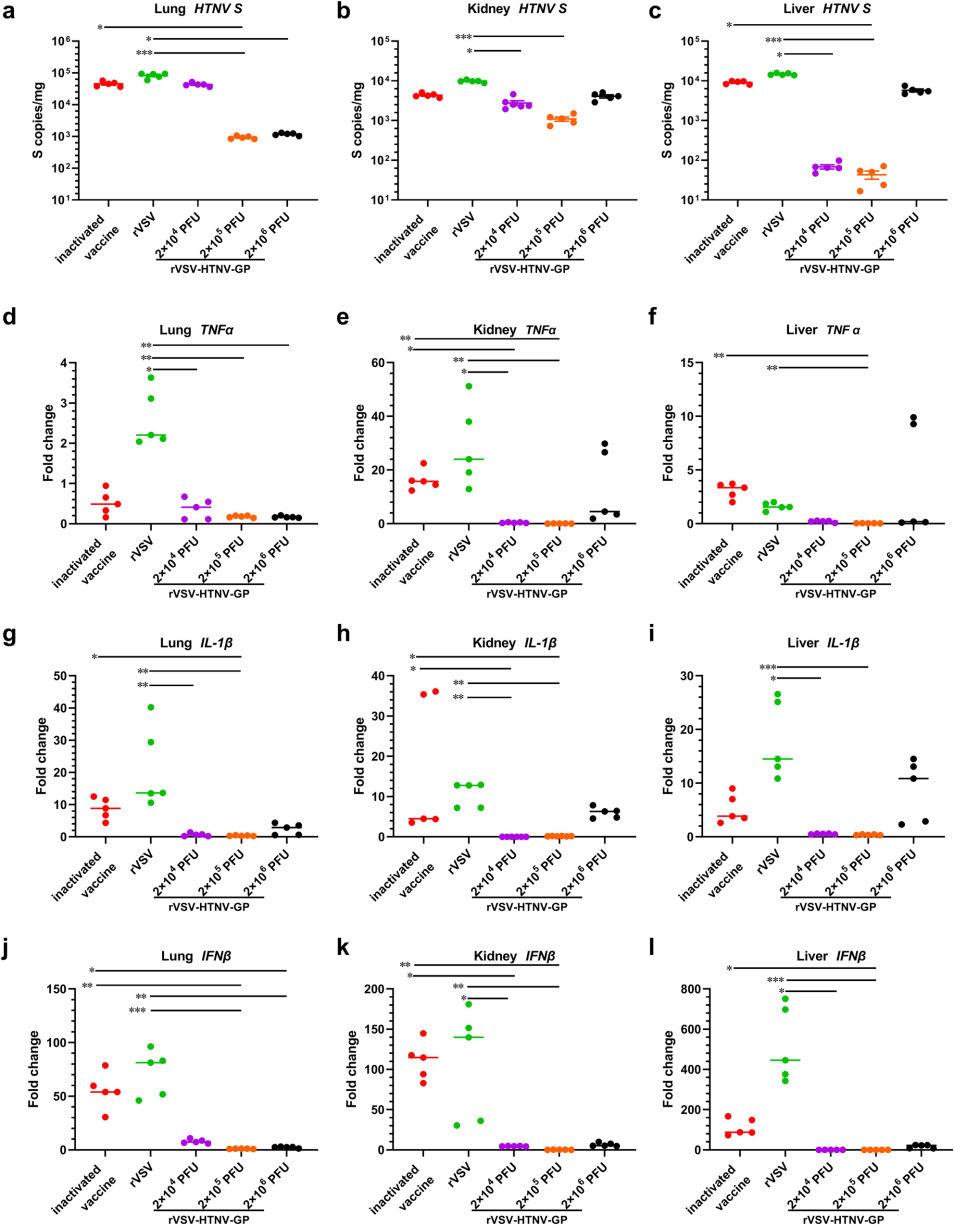
rVSV-HTNV-GP vaccination reduces viral loads in multiple tissues and protects from Virus-Induced Inflammation. **a**–**c** Four weeks after rVSV or rVSV-HTNV-GP immunization, or three weeks after the last boost of inactivated vaccine, mice were challenged with 1 × 10^6^ FFUs of HTNV via intramuscular administration. Three days later, tissues were harvested, and the determination of viral burden in the lung (**a**), kidney (**b**), and liver (**c**) by RT-qPCR assay. Cytokine TNFα in mice lung (**d**), kidney (**e**), and liver (**f**) tissues evaluated by RT-qPCR assay. Cytokine IL-1β in mice lung (**g**), kidney (**h**), and liver (**i**) tissues evaluated by RT-qPCR assay. Cytokine IFNβ in mice lung (**j**), kidney (**k**), and liver (**l**) tissues evaluated by RT-qPCR assay. Data are shown as fold change in gene expression compared to fully naive, age-matched animals after normalization to GAPDH. (*n* = 5 mice per group; data of viral loads are represented as mean ± SEMs, and data of cytokine are represented as median values. Kruskal–Wallis test with Dunn’s post-test: *P < 0.05, **P < 0.01, ***P < 0.001, ****P < 0.0001).

To test whether rVSV-HTNV-GP elicited higher levels of NAbs to protect against HTNV infection, the mice were challenged with HTNV, and euthanized 3 days post infection (Fig. 3a). The viral RNA burdens were determined using quantitative reverse transcription polymerase chain reaction (PCR) (RT–qPCR) in mouse lung, kidney, liver, and spleen tissues. Viral RNA was markedly reduced in the rVSV-HTNV-GP groups, compared to the rVSV and inactivated vaccine groups, and was undetectable in 2 × 10^5^ PFUs of rVSV-HTNV-GP groups, suggesting sterile protection (Fig. 4a–c, Supplementary Fig. 5g).

The pro-inflammatory cytokine messenger RNA (mRNA) levels in homogenates of lung, kidney, liver, and spleen tissues from vaccinated animals at 3 dpi were measured using RT-qPCR to assess whether rVSV-HTNV-GP limited HTNV-induced inflammation (Fig. 3a). Mice immunized with different doses of rVSV-HTNV-GP had significantly lower levels of proinflammatory cytokine mRNA as opposed to rVSV-and inactivated vaccine-immunized mice. Specifically, TNFα (Fig. 4d–f, Supplementary Fig. 5h), IL-1β (Fig. 4g–i, Supplementary Fig. 5i), IL-6 (Supplementary Fig. 5a–c, k), IL-10 (Supplementary Fig. 5d–f, l), and type I interferons (IFN-β) (Fig. 4j–l, Supplementary Fig. 5j) decreased early during infection in all three regimes of mice immunization with rVSV-HTNV-GP. Although there were no detectable differences among the three rVSV-HTNV-GP groups, the 2 × 10^5^ PFUs group performed the best, followed by 2 × 10^4^ PFUs. Altogether, these data establish that immunization with rVSV-HTNV-GP protects against HTNV infection in mice, and that NAbs are a possible protective factor.

### rVSV-HTNV-GP vaccination reduces pathological damage in multiple tissues against HTNV Infection

**Fig. 5:**
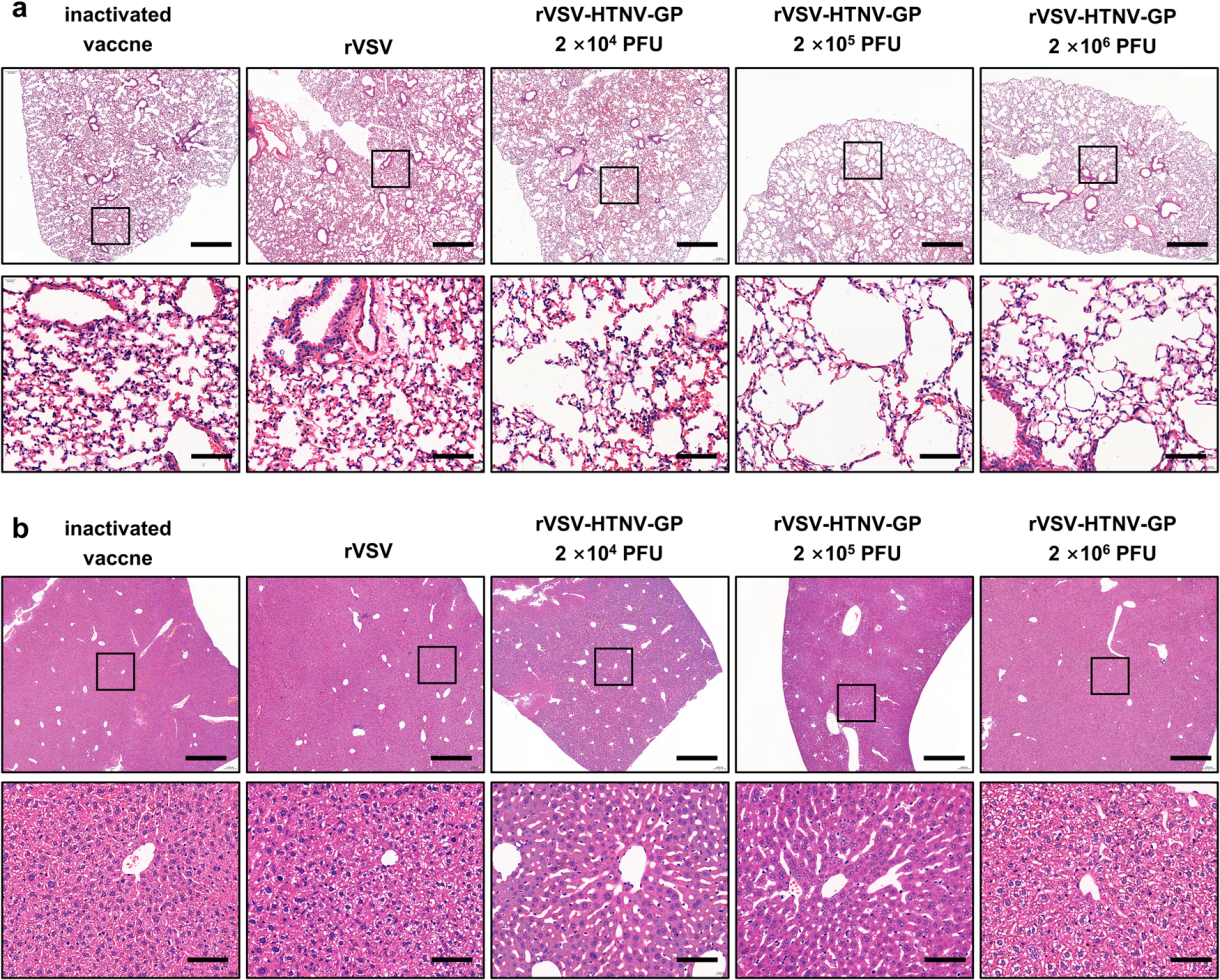
rVSV-HTNV-GP Protects Mice from HTNV induced pathological injury. Hematoxylin and eosin staining of lung (**a**) and liver (**b**) sections from immunized mice at 3 dpi from 1 × 10^6^ FFUs of HTNV challenge. (upper scale bars, 80 μm; lower scale bars, 10 μm).

The extent of tissue damage in HTNV-challenged mice was determined by staining lung and liver sections with hematoxylin and eosin (H&E) (Fig. 5a, b). Lung sections from rVSV-HTNV-GP-immunized mice, especially the 2 × 10^5^ and 2 × 10^6^ PFUs groups, showed the lowest signs of inflammation (Fig. 5a), which matched the viral RNA levels in the corresponding tissues (Fig. 4a). Shrinkage of alveolar tissue and immune cell infiltration was observed in rVSV-immunized mice (Fig. 5a). Hepatocellular edema and dilated and congested hepatic sinusoids were documented in the rVSV-, inactivated vaccine- and 2 × 10^6^ PFUs of the rVSV-HTNV-GP-immunized mice groups, but not in the 2 × 10^4^ and 2 × 10^5^ PFUs of the rVSV-HTNV-GP groups (Fig. 5a). This was consistent with the viral burden in the corresponding tissues (Fig. 4c). Thus, immunization with rVSV-HTNV-GP generates a protective immune response, particularly at a dose of 2 × 10^5^ PFUs, thereby limiting HTNV-induced lung and liver inflammation in mice.

### Immunization with a single-dose regime of rVSV-HTNV-GP induced neutralizing antibodies is superior to the two-doses regime

**Fig. 6:**
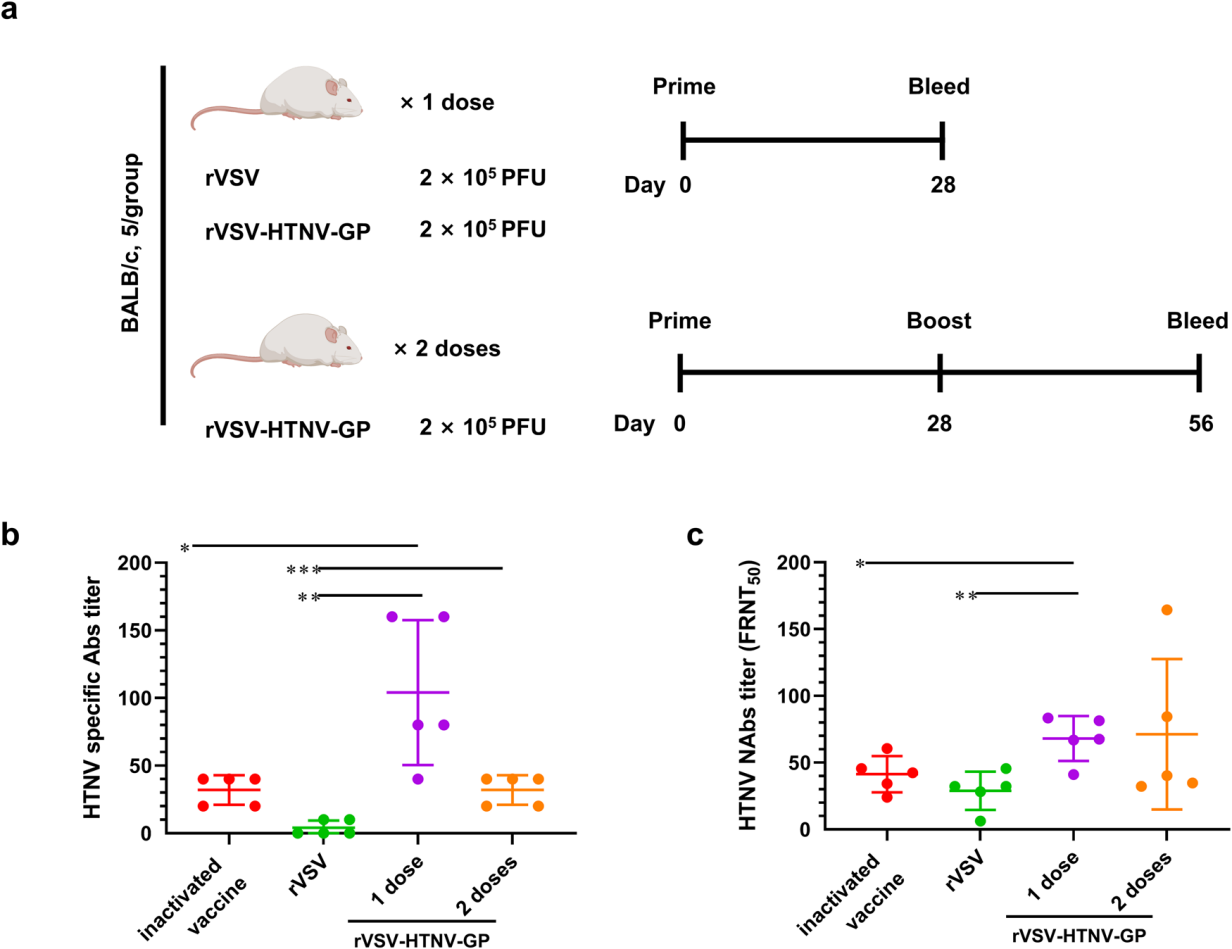
Prime-boost immunization regimen of rVSV-HTNV-GP didn’t improve the immunological index compared to a single dose. **a** Scheme of prime-only and prime-boost vaccination regimens. For the prime-only regime, 6-week-old female BALB/c mice were immunized with 2 × 10^5^ PFUs of rVSV or rVSV-HTNV-GP, and the HFRS-inactivated vaccine was used as a positive control. For the prime-boost regimen, rVSV-HTNV-GP mice were immunized twice at a 4-weeks interval. The inactivated vaccine group was boosted twice with a 3-weeks interval, after the primary vaccination. **b** HTNV specific IgG responses in the sera of vaccinated mice evaluated 4 weeks after priming or boosting by IFA for binding to HTNV for rVSV or rVSV-HTNV-GP group. For inactivated vaccine group, serum sample were collected 3 weeks after last boost. **c** Neutralizing antibody titers against HTNV determined by FRNT. (For **b** and **c**, *n* = 5 mice per group; data are represented as the means ± SEMs, one-way ANOVA with Dunnett’s post-test: *P < 0.05, **P < 0.01).

Given that the immunization regime with 2 × 10^5^ PFUs of rVSV-HTNV-GP showed the best performance in inducing NAbs and limiting tissue inflammation response, the immunization regime had to be standardized for the number of doses. Therefore, a second booster dose of rVSV-HTNV-GP was administered to determine if NAb titer against HTNV improved significantly (Fig. 6a). HTNV-specific antibodies and NAb titers against HTNV were measured after priming or boosting. Out of expected, boosting failed to significantly enhance HTNV-specific antibodies or NAb titers (Fig. 6b, c). Immunization with rVSV-HTNV-GP induced higher levels of anti-GP-specific IgG compared to the rVSV control, with reciprocal median serum endpoint titers of 104.0 and 32.0 for one and two doses of the vaccine, respectively (Fig. 6b). The NAb titers of the single- and two-dose immunization regimes of rVSV-HTNV-GP were 68.0 and 71.0, respectively, whereas that of the inactivated vaccine was 41.0 (Fig. 6c). Boosting failed to increase the HTNV-specific antibody titer after the second dose of rVSV-HTNV-GP, whereas the NAbs titer enhancement was limited and not statistically significant. These data suggest that rVSV-HTNV-GP elicits high titers of both HTNV-specific antibodies and NAbs in a single dosage.

### Single-dose of rVSV-HTNV-GP immunization elicits durable neutralizing antibodies response

**Fig. 7:**
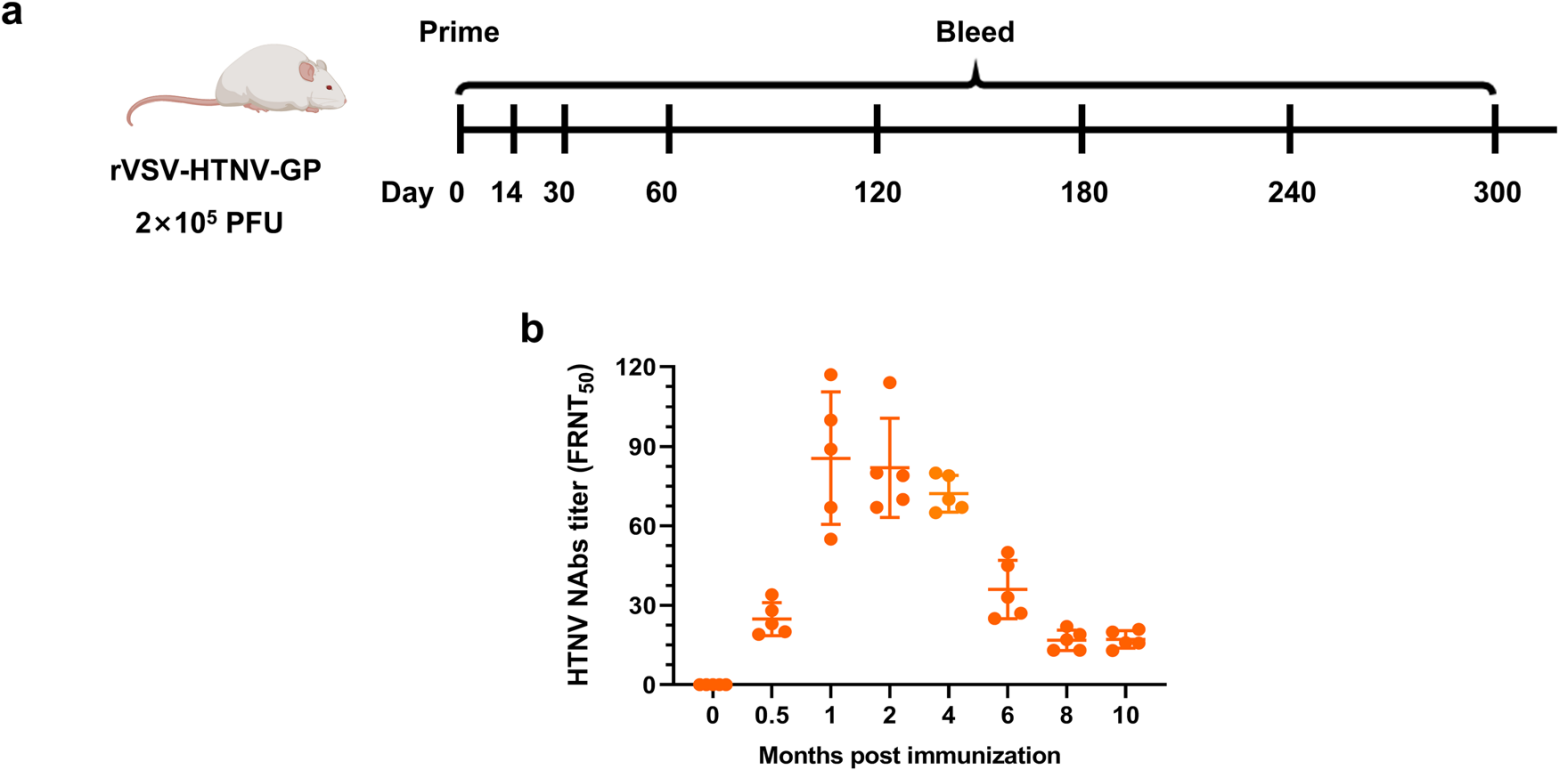
Single dose of rVSV-HTNV-GP immunization elicits long-lasting neutralizing antibodies response in mice. **a** Vaccination scheme. Six-week-old female BALB/c mice immunized with 2 × 10^5^ PFUs of rVSV-HTNV-GP and serum samples from each mouse harvested at 0, 14, 30, 60, 120, 180, 240, and 300 days post immunization. **b** Neutralizing antibody titers against HTNV determined by FRNT. (*n* = 5 mice per group; data are represented as the means ± SEMs, one-way ANOVA with Dunnett’s post-test: *P < 0.05, **P < 0.01).

As a single dose of rVSV-HTNV-GP immunization elicited high levels of NAbs and boosting failed to enhance the NAbs titer, we speculated that priming induces a durable NAbs response that compensates for the second dose of rVSV-HTNV-GP. To test this hypothesis, we immunized a group of BALB/c mice with a single dose of 2 × 10^5^ PFUs of rVSV-HTNV-GP and collected blood samples at 0, 0.5, 1, 2, 4, 6, 8, and 10 months post immunization, respectively (Fig. 7a). The NAbs were then measured using FRNT. The NAbs titer gradually increased after immunization and reached a peak (85.6) at 1 month post immunization, and was maintained at a high level between 2 and 4 months (82.0 and 72.2, respectively). This was followed by a gradual decrease (36.0 at 6 months) (Fig. 7b). Ten months post immunization, the NAb titer remained detectable at 17.0 (Fig. 7b). Thus, rVSV-HTNV-GP elicited high titers of NAb that were maintained at the peak level for 3 months and were sustained at a detectable level for over 10 months. This suggests that long-lived plasma cells were induced after rVSV-HTNV-GP immunization.

### Single-dose of rVSV-HTNV-GP immunization provides long-term protection against HTNV infection

**Fig. 8:**
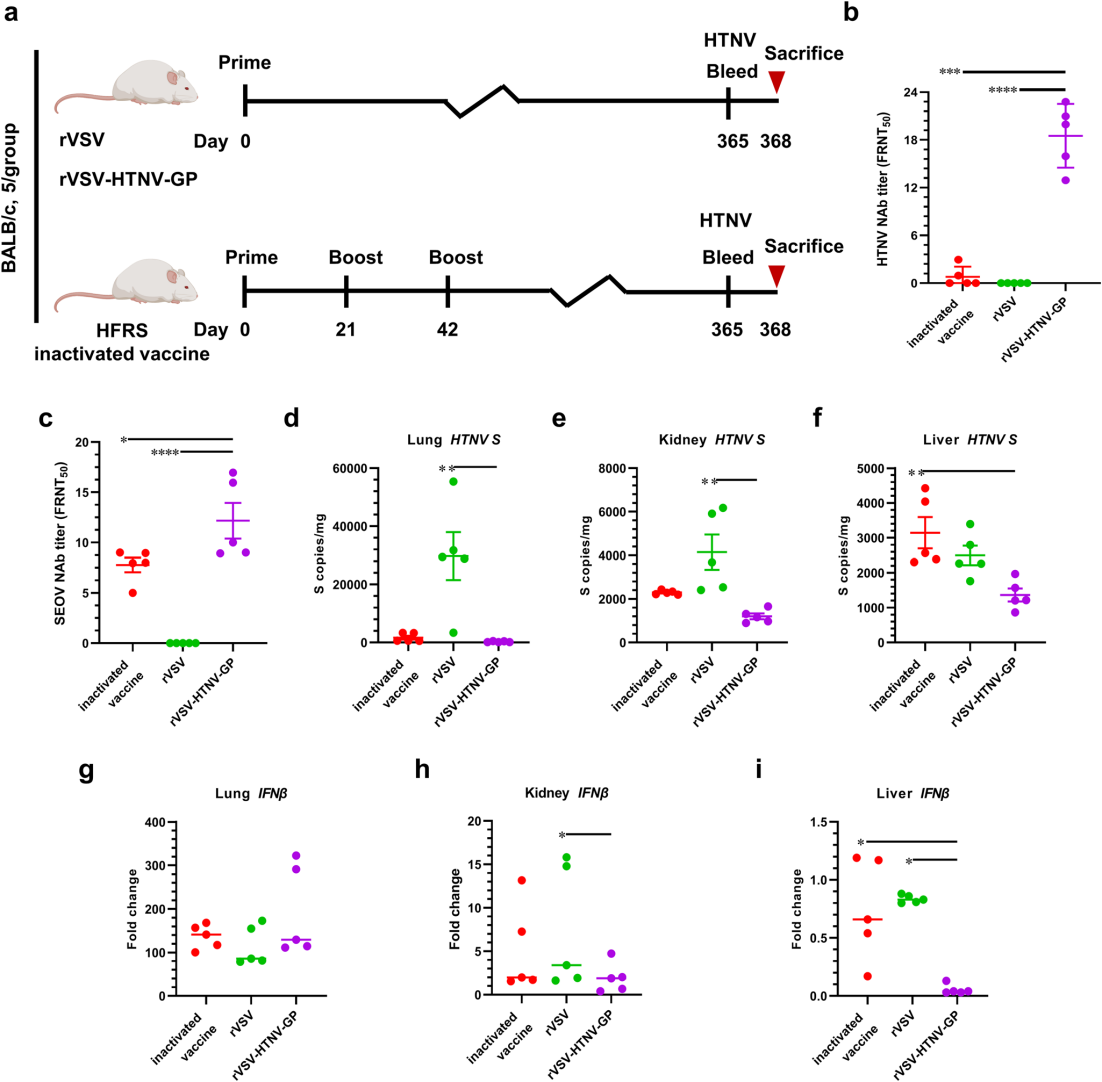
Single dose of rVSV-HTNV-GP immunization provides long-lasting protection to mice. **a** Scheme for vaccination and HTNV challenge. Six-week-old female BALB/c mice were immunized with 2 × 10^5^ PFUs of rVSV or rVSV-HTNV-GP. The HFRS-inactivated vaccine was used as a positive control. For the inactivated vaccine group, the mice were boosted twice at 3-week intervals. One year later, the mice were intramuscularly challenged with 1 × 10^6^ FFUs of HTNV. **b**, **c** Long term neutralizing antibody titer. One year after immunization with rVSV, rVSV-HTNV-GP, or an inactivated vaccine, mouse serum was harvested, and neutralizing antibody titers against HTNV (**a**) and SEOV (**c**) were determined using FRNT. (*n* = 5 mice per group; data are represented as the means ± SEMs; one-way ANOVA with Dunnett’s post-test: *P < 0.05, ***P < 0.001, ****P < 0.0001). One year after immunization, the mice were challenged with 1 × 10^6^ FFUs of HTNV intramuscularly. Determination of viral loads in the lungs (**d**), kidneys (**e**), and liver (**f**) of immunized mice, 3 days post challenge, 1 year after immunization by RT-qPCR. Data are shown as fold change in gene expression compared to fully naive, age-matched animals after normalization to GAPDH. Evaluation of cytokine IFNβ in mice lung (**g**), kidney (**h**), and liver (**i**) tissues by RT-qPCR assay. For **d**–**I**, data are shown as fold-change in gene expression compared to fully naïve, age-matched animals after normalization to GAPDH. (*n* = 5 mice per group; data are represented as means ± SEMs; Kruskal–Wallis test with Dunn’s post-hoc test: *P < 0.05, **P < 0.01).

Twelve months post rVSV-HTNV-GP immunization, blood samples were collected from each group (Fig. 8a). The NAb titers were measured; they were undetectable in the rVSV group, whereas the NAb titer in the three sequential injections of inactivated vaccines was only 0.7832, as compared to 18.52 in the rVSV-HTNV-GP group (Fig. 8b). Additionally, the level of SEOV crossing NAb in the rVSV-HTNV-GP group was higher than that in the inactivated vaccine-immunized mice (12.97 and 9.383, respectively). Given that a single dose of rVSV-HTNV-GP immunization maintained a relatively high NAb titer after 1 year, whether this level of NAb provided protection against HTNV challenge was evaluated. Mice were challenged intramuscularly with 1 × 10^6^ FFUs of HTNV to evaluate vaccine protection. The viral loads in the lungs and kidneys were significantly lower than those in the rVSV group (Fig. 8d–f), whereas the viral load in the rVSV-HTNV-GP group was lower than that in the inactivated vaccine group, although the differences were not significant (Fig. 8d–f). Subsequently, inflammatory cytokine levels in different organs were measured using RT-qPCR. The IFN-β in the lung and kidney was not significantly different between inactivated vaccines, rVSV, and rVSV-HTNV-GP groups (Fig. 8g, h); however, the liver IFN-β was lower in rVSV-HTNV-GP group than in inactivated vaccines and rVSV groups (Fig. 8i). Moreover, the TNFα and IL-6 were both lower in rVSV-HTNV-GP group than in rVSV groups (Supplementary Fig. 6a–c, g–i). In addition, kidney IL-1β was lower in rVSV-HTNV-GP group, while inflammatory factors were not significantly different in lungs between groups (Supplementary Fig. 6a–l). Nonetheless, single dose of rVSV-HTNV-GP immunization provided better protection than the rVSV group and provided similar inflammatory cytokine responses compared to the three sequential injections of inactivated vaccines to some extent.

## Discussion

*Bunyavirales* comprise multiple highly pathogenic viruses, including Crimean–Congo hemorrhagic fever virus (CCHFV), Rift Valley fever virus (RVFV), severe fever with thrombocytopenia syndrome virus (SFTSV), and hantaviruses^22–24^. Among these, hantaviruses is the most widely distributed. HTNV caused HFRS, exhibiting the highest mortality among other OWHs^1^. Suckling mouse brain-based HTNV monovalent inactivated vaccines (Hantavax^TM^) have been licensed in South Korea for decades, while HTNV and SEOV bivalent inactivated vaccines have been used in endemic areas in China for years^25,26^. Although protective, the NAbs induced by inactivated vaccines are quite low and quickly disappear^10^. Moreover, complicated vaccination regimes are associated with poor adherence in rural areas. Thus, there is an urgent need to develop an effective HTNV vaccine that can enhance its ability to induce NAbs and simplify its administration regime. Therefore, the development of a single-dose immunization regime that induces high levels of NAbs is urgently needed. This study showed that rVSV based rVSV-HTNV-GP elicited high levels of NAbs with only a single dose in a mouse model and provided sterile protection against HTNV challenge.

Numerous vectored platforms have been investigated in animal models against hantavirus infection. Vaccinia virus-, adenovirus-, and lentivirus-vectored HFRS vaccine candidates have been extensively investigated by different groups^12,13,27^. However, improvements in NAbs induced by these candidates is limited. One reason for this is that the hantavirus GP is encoded within the viral vector genome and is expressed only upon vector infection. The pre-existing immunity against the vector also poses other obstacles to the clinical use of these candidates^28^. rVSV is an ideal vaccine platform that can easily incorporate other viral GPs and has been investigated extensively^16,29–31^. The most evident example is Merck’s certified ERVEBO, which is a replication-competent VSV that encodes the EBOV glycoprotein and shows the ability to induce a humoral immune response^32^.

As a replication-competent vector, an rVSV-based vaccine can be easily amplified for bulk manufacturing. Safety is also a key consideration in vaccine development, even though human infections with VSV are extremely rare, with only a limited number of reports^33–35^. The main pathogenic factor of VSV is VSV-G, which is substituted with foreign GPs in case of the vectored vaccine. The negative-sense single-stranded RNA genome of VSV is not prone to integration into the host genome or genetic reassortment, enhancing its safety when used in humans. In addition, there is nearly no pre-existing immunity to VSV in humans; hence, it would not interfere with efficacy of foreign glycoproteins expressing-rVSV^36^.

Furthermore, rVSV-HTNV-GP produced cross-reactive NAbs against both HTNV and SEOV. Previous studies have consistently revealed that this phenomenon exists within NWHs because rVSV-Andes *orthohantavirus* (ANDV) can provide cross-protection against both ANDV and Sin Nombre viruses, and vice versa^37^. Testing of the long-term immunogenicity and protection provided by a single rVSV-HTNV-GP vaccination revealed that both HTNV and SEOV NAbs were detectable after 1 year. As there is a lack of a lethal small animal model for OWHs, the main limitation of this study was attributed to the inflammation and viral loads of the different tissues induced by HTNV challenge, instead of animals that succumbed to infection.

Notably, from days 30–120 post vaccination, these mice had similar HTNV-specific NAb titers which sustained at the peak titers, followed by a drop by half on day 180, suggesting that the second boost dose may achieve the best results at least 6 months after priming. Consistent with our two-dose regime data, in which the boost dose was administered at 4-weeks post priming, and no significant improvement was observed compared to that of the single-dose regime.

Although a single injection of rVSV-HTNV-GP provides long-term protection from HTNV infection, improvements compared to three sequential injections of inactivated vaccines is limited. HTNV NP in the formulation may lead to non-inferior protection of the inactivated vaccine. Numerous studies have suggested that hantavirus NP is a protective antigen and strong T-cell immunity activator^27,38–40^. Nevertheless, a recent study inserted both a severe acute respiratory syndrome coronavirus 2 (SARS-CoV-2) spike and NP into the rVSV genome and rescued the recombinant virus, which showed improved protective efficacy in a hamster model^41^. Further investigation is required to determine whether the addition of hantavirus NP to the VSV genome can augment rVSV-HTNV-GP-induced protection.

**Fig. 9:**
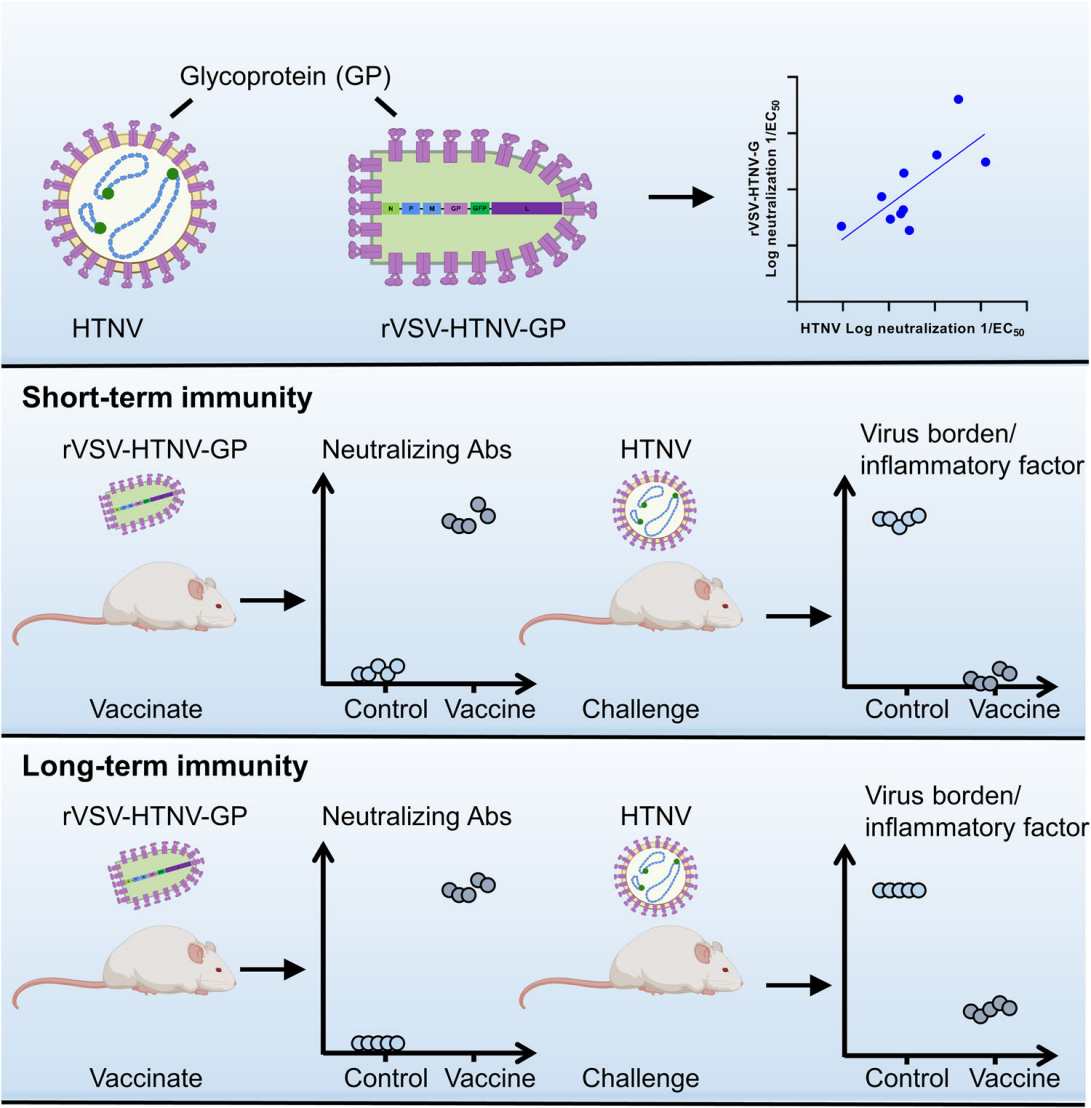
Working model showing rVSV-HTNV-GP immunization provides protection to mice by eliciting NAbs.

In summary, we determined that recombinant VSV expressing the HTNV GP could elicit robust, durable, and OWH cross-reactive NAbs in a mouse model (Fig. 9). It also provides long-term protection from HTNV infection, which is promising for further evaluation in non-human primate models and clinical development.

## Methods

### Cells, viruses, and antibodies

Baby hamster kidney cells (BHK-21), African green monkey kidney epithelial cells (Vero E6), human non-small-cell lung carcinoma cells (A549), human hepatoma cells (Huh7), and human cervical carcinoma (HeLa) cells were maintained in Dulbecco’s Modified Eagle Medium (DMEM) (Corning, NY, USA) with 10% fetal bovine serum (FBS) (YEASEN, Shanghai, China) and 1% penicillin-streptomycin-gentamicin solution (Solarbio, Beijing, China). HUVECs were maintained in an endothelial cell medium (ECM) (ScienCell, CA, USA). All cells were maintained in humidified incubators at 37 °C supplemented with 5% CO_2_. HTNV (strain 76-118) and SEOV (strain SR-11) were stored in our laboratory.

Mouse monoclonal antibody (mAb) 1A8 against HTNV NP, mAb Gn-1 against HTNV Gn, and mAb Gc-10 against HTNV Gc were produced in our lab as previously indicated^42^. The anti-VSV-G tag was purchased from abcam (Cambridge, UK). Infrared (IR)Dye680RD-conjugated goat anti-mouse and anti-rabbit secondary antibodies were purchased from Lambda Instruments Corporation (LI-COR) Biosciences (LI-COR, NE, USA). Cyanine3 (Cy3)-conjugated goat anti-rabbit IgG, goat anti-mouse IgG, fluorescein isothiocyanate (FITC)-conjugated goat anti-mouse IgG, and horseradish peroxidase (HRP)-conjugated anti-mouse IgG were purchased from Biotech & Bio Basic Inc. (BBI) (Sangon Biotech). Hoechst 33258 stain was purchased from YEASEN.

Monoclonal Antibodies used for flow cytometry analysis, eFluor 506-CD3, FITC-CD4, phycoerythrin (PE)-IL-4, PE-Cyanine7-CD8a, allophycocyanin (APC)-IFNγ, Peridinin-Chlorophyll-Protein (PerCP)-eFluo 710-TNFα and Fixable Viability Dye eFluo 780 organic dye (FVD-780) were all obtained from eBioscience (Massachusetts, USA).

### Recombinant VSV recovery and titration

The plasmid-bearing VSV antigenome lacking VSV-G ORF but with an additional GFP ORF was synthesized at GenScript (Nanjing, China). The generation and production of rVSV and rVSV-HTNV-GP was performed as previously described^43^. An infectious cDNA clone of VSV was engineered by replacing endogenous VSV-G with HTNV GP and addition of a fluorescent marker (GFP) between the HTNV GP and VSV-L ORFs. To facilitate rVSV rescue, the endoplasmic reticulum (ER) retention sequence in the HTNV Gc cytoplasmic tail was truncated to alter HTNV GP retargeting to the plasma membrane instead of the ER-Golgi intermediate compartment (ERGIC)^44^. To further enhance rescue efficiency, we mutated leucine 532 to lysine (I532K) and serine 1094 to leucine (S1094L). S1094L was located within the viral membrane-proximal external region (MPER), which would increase the amphipathic character of Gc MPER (designated as rVSV-HTNV-GP)^45,46^. For rVSV-HTNV-GP, the codon-optimized GPC gene of HTNV (NC_005219) was amplified using PCR and inserted into the VSV antigenome plasmid, resulting in the rVSV-HTNV-GP vector. BHK-21 cells were inoculated with a vaccinia virus bearing T7 RNA polymerase (VV-T7), kindly provided by the Wuhan Institute of Virology, CAS, followed by co-transfection of five plasmids: full-length rVSV-HTNV-GP, together with VSV accessory plasmids encoding VSV-N, VSV-P, VSV-L, and VSV-G proteins, all of which were under T7 promoter control. The culture supernatant was collected 72 h post transfection and used to infect Vero E6 cells. The construct was recovered following an established protocol^15,47^. After several passages, the cytopathic effect (CPE) in the cell monolayer was noticeable, indicating a successful rescue. The rescued virus was referred to as rVSV-HTNV-GP and verified by HTNV GPC-specific antibodies.

The viruses were propagated and titrated on Vero E6 cells, and the titer was determined using plaque assays. Briefly, Vero E6 cells were inoculated into 12-well plates and grown overnight until the cells were more than 90% confluent. The stock of rVSV and rVSV-HTNV-GP were 10-fold serially diluted and used to infect Vero E6 cells at a concentration of 500 μL per well in triplicates. After incubation at 37 °C for 2 h, the supernatant was aspirated and 2 mL maintenance overlay DMEM supplemented with 2% FBS and 2% carboxymethyl cellulose (CMC) was added to each well. The plates were then incubated for the desired time (3 days for rVSV and 7 days for rVSV-HTNV-GP). The overlay medium was aspirated, and the cells were stained with crystal violet staining solution (Beyotime, Shanghai, China) for 30 min at room temperature. The number of plaques in each well was counted and the titers of rVSV and rVSV-HTNV-GP were calculated.

### Growth kinetics of rVSVs

Vero E6 cells in 6-well plates at 90% confluency were infected in triplicates with rVSV or rVSV-HTNV-GP at a multiplicity of infection (MOI) of 0.0001. Following incubation at 37 °C for 2 h, the plates were washed three times and the culture media was replaced with DMEM supplemented with 2% FBS and cultured at 37 °C with 5% CO_2_. The culture supernatant was collected from each well at 0, 12, 24, 48, 60 and 72 hours post infection (hpi), and subjugated to TCID_50_ analysis. In short, ten-fold serial dilutions of each supernatant were added to 96-well plate in quadruplicate. After removing the virus, the maintenance overlay medium was added. At 7 dpi, the viscous overlay medium was discarded, the cells were stained with crystal violet solution, and the TCID_50_ values were then calculated using the Reed-and-Muench method.

### Virus purification and transmission electron microscopy

rVSV and rVSV-HTNV-GP were propagated in Vero E6 cells. The culture supernatant was harvested and clarified by centrifugation at 5000 × *g* for 30 min at 4 °C. The supernatant was then concentrated by mixing with four times the volume of 0.8 M NaCl buffered 5 × polyethylene glycol (PEG) 8000 (Sigma-Aldrich, St. Louis, USA) and incubated at 4 °C overnight, followed by centrifugation at 10,000 × *g* for 1 h at 4 °C. The pellet was then re-suspended in 1 mL Tris-HCl (Tris-HCl: 0.5 mM Tris-HCl, 1.5 mM NaCl, 0.05 mM ethylenediaminetetraacetic acid (EDTA), and H_2_O 1 mL) at 4 °C overnight.

The resuspended solution was then loaded onto an iodixanol (Sigma-Aldrich) density gradient medium, which was formed by underlaying 4.2 mL of 15% iodixanol solutions onto 3.8 mL 35% iodixanol solutions. The viruses were banded by ultracentrifugation at 160,000 × *g* for 2 h at 4 °C using an SW41 Ti rotor (Beckman Coulter, Brea, USA). The purified virus adhered to glow-discharged carbon-coated copper grids. The samples were stained with 4% (w/v) phosphotungstic acid (pH 7.1) and viewed under a JEM-1400 Flash transmission electron microscope (JEOL, Japan).

### SDS-PAGE and western blotting

Purified rVSV and rVSV-HTNV-GP were mixed with 4 × lithium dodecyl sulfate (LDS) sampling buffer (GenScript) and boiled at 100 °C for 10 min. Viral proteins were examined using SDS-PAGE followed by Coomassie Brilliant Blue G-250 (YEASEN) staining. For western blotting, viral proteins were separated and transferred onto a Poly (vinylidene fluoride) membrane, and incubated with mouse anti-Gn-1 (1:100), mouse anti-Gc-10 (1:1000), or rabbit anti-VSV-G (1:2000, abcam, 309106) antibody diluted in Tris-buffered saline containing 1% Tween-20 (TBST), followed by incubation with IRDye 680RD Goat anti-Mouse (1:10000, LI-COR, 926-68070) or IRDye 680RD Goat anti-Rabbit (1:10000, LI-COR, 926-32211), and imaged with an Odyssey Imager (LI-COR).

### Immunofluorescence assay

Vero E6 cells were seeded in 24-well plates and incubated at 37 °C overnight. HTNV, rVSV, or rVSV-HTNV-GP was used to infect cells for 2 h, followed by medium change to DMEM containing 2% FBS and cultured for an additional 48 h. Cells were fixed with 4% paraformaldehyde (PFA) at room temperature for 15 min, permeabilized with 0.5% Triton X-100 for 10 min, and stained with 1A8 (HTNV NP) (1:1000), anti-Gn-1 (1:50), anti-Gc-10 (1:100), or anti-VSV-G (1:1000, abcam, 309106) antibodies at 4 °C overnight. After washing, Cy3-conjugated goat anti-mouse IgG (1:400, Sangon Biotech, D111024) or Cy3-conjugated goat anti-rabbit IgG (1:400, Sangon Biotech, D111018) were added to each well and incubated at room temperature for 1 h. The nuclei were visualized using Hoechst 33258 (1:1000, YEASEN, 40729ES10). The images were captured using a BX60 fluorescent cell imager (Olympus, Tokyo, Japan).

### Focus-reduction neutralization test (FRNT)

The FRNT assay was performed according to a previously established protocol with mild modifications^21^. Vero E6 cells were seeded in 96-well plates and cultured until the cell confluency was greater than 90%. Ten convalescent serum samples from patients with HFRS were serially diluted (between 1:20 and 1:43,740) in 200 µL of infection medium, mixed with the same volume of 200 focus forming units (FFUs) of HTNV, and incubated at 37 °C for 1 h. The convalescent serum sample-related experiments were approved by the Ethics Committee of Tangdu Hospital, Air Force Medical University (approval number:202103-139). Cells were then infected with serum-virus complexes with a concentration of 100 μL/well in quadruplicates and adsorbed at 37 °C for 2 h. After removing the serum-virus complexes, each well was covered with a maintenance overlay medium. The overlay medium was discarded at 5 dpi, and the plate was fixed, permeabilized, and blocked with 3% bovine serum albumin (BSA) (YEASEN). mAb 1A8 (1:1000) was used to probe the HTNV NP at 4 °C overnight. After discarding 1A8, HRP-conjugated goat anti mouse IgG (1:1000, Sangon Biotech, D110087) was added, and the cells were incubated for 1 h at room temperature. After washing thrice, the plate was stained with 3, 3’, 5, 5’-tetramethylbenzidine substrate for blotting (Biokits, Beijing, China) for 30 min at 37 °C. The plate was then dried, and the foci were counted. The Nab titer was calculated. The FRNT_50_ was defined as the maximum dilution of serum that inhibited HTNV infection in 50% of the cells. For mouse NAb detection, the same method was adopted, but mouse sera were diluted 1:10, 1:20, 1:40, 1:80, and 1:160 times.

### GFP-based foci-reduction neutralization test

The indicated dilutions of HFRS convalescent serum samples were incubated with 100 plaque forming units (PFUs) of rVSV-HTNV-GP for 1 h at 37 °C. Serum-virus complexes were added to Vero E6 cells in 96-well plates and incubated at 37 °C for 7 h. Subsequently, cells were fixed in 4% PFA and stained with Hoechst 33258. Images were acquired with a BioTek Cytation 5 imaging reader (Agilent, California, USA) in FITC channel to visualize GFP-positive cells. Data were processed using Prism software (GraphPad 8.0, Inc., La Jolla, CA, USA).

### Mouse experiments and ethics statement

Six-to-eight-week-old female BALB/c mice were provided by the Laboratory Animal Centre of Air Force Medical University. All animal studies were carried out in strict accordance with the recommendations in the Guide for the Care and Use of Laboratory Animals of the Ministry of Science and Technology of the People’s Republic of China. The protocols for animal studies were approved by the Committee on the Ethics of Laboratory Animal Centre of Air Force Medical University (Approval number: 20200403). HTNV challenge experiments were performed in a biosafety level 3 containment facility.

### Short-term immunization regime

The BALB/c mice were randomly divided into five groups (*n* = 5 per group). Mice were intraperitoneally (i.p.) injected with the HFRS-inactivated vaccine, rVSV, or rVSV-HTNV-GP, and 200 μL (100 μg) HFRS inactivated vaccine was administered three times at 3 week intervals. Then, 2 × 10^4^ PFUs of rVSV were administered. For rVSV-HTNV-GP, three different doses of 2 × 10^4^, 2 × 10^5^, or 2 × 10^6^ PFUs were administered. Tail bleeds were taken from on 4 weeks after rVSV or rVSV-HTNV-GP administration, and 3 weeks after the vaccination regime was completed. HTNV-specific antibody and NAb levels were determined based on serum samples. HTNV-specific antibody titers were determined by immunofluorescence assay (IFA). Specifically, HTNV-infected Vero E6 cells in 48-well plates were probed with two-fold serial dilutions of immunized mice sera, and FITC-conjugated anti-mouse IgG (1:400, Sangon Biotech, D110105) was applied. The titer of specific antibodies was calculated using the maximum valid double dilution, while still being positive for HTNV. The titer of the HTNV-neutralizing antibody was determined based on FRNT. After bleeding, the animals were euthanized by cervical vertebra dislocation following isoflurane (RWD, R510-22-10) anesthesia, spleen cells were collected, and cytokines in CD4^+^ T cells were evaluated using flow cytometry.

To assess the protective effect, mice in each group were intramuscularly (i.m.) challenged with 1 × 10^6^ FFUs of HTNV. Animals were sacrificed at 3 dpi and viral loads in the liver, lung, spleen, and kidney were determined using qRT-PCR. The mRNA levels of inflammatory cytokines, including *IL-1β, IL-6, IL-10, TNFα*, and *IFNβ*, were also determined in these tissues.

### Prime-boost immunization regime

The BALB/c mice were randomly divided into four groups (*n* = 5 per group). Mice were i.p. injected with HFRS-inactivated vaccine, rVSV, or rVSV-HTNV-GP. For one dose regime, 2 × 10^5^ PFUs of rVSV or rVSV-HTNV-GP were administered. For two dose regime, 2 × 10^5^ PFUs of rVSV-HTNV-GP were administered twice at a 4-week interval. After vaccination, tail bleeds were taken, and HTNV-specific antibodies and NAbs were evaluated.

### Long-term immunization regime

Mice were immunized with 2 × 10^5^ PFUs of rVSV or rVSV-HTNV-GP via an i.p. route. In the inactivated vaccine group, the vaccines were administered thrice at a 3-week interval. Tail bleeds were taken from the rVSV-HTNV-GP-vaccinated mice at 0, 14, 30, 60, 120, 160, 240, and 300 dpi. One year after immunization, tail bleeds were taken from all mice, and NAbs were measured. The mice were intramuscularly injected with 1 × 10^6^ FFUs of HTNV and euthanized at 3 dpi. Viral loads and inflammatory cytokine mRNA levels in the liver, lung, and kidney were evaluated.

### RT-qPCR-based viral burden measurement and cytokine analysis

Mouse tissues were weighed and homogenized using sterile stainless-steel beads in a TissueLyser II instrument (QIAGEN, Germany) in 1 mL of DMEM supplemented with 2% FBS. Total RNA was extracted from clarified tissue homogenates using Total RNA Isolation (TRizol) Universal RNA Extraction kit (TIANGEN, China). Further, 1 μg of total RNA was used to generate cDNA using the Hifair II 1st Strand cDNA Synthesis Kit (YEASEN) as per manufacturer’s instructions. Viral RNA levels were determined by RT-qPCR using Hieff qPCR SYBR Green Master Mix (YEASEN), as described previously, using the primer pair HTNV-S (forward:5′-GAGCCTGGAGACCATCTG-3′; reverse:5′-CGGGACGACAAAGGATGT-3′)^8^.

The mRNA levels of the inflammatory cytokine were determined using specific primer pairs for IL-1β, IL-6, IL-10, TNFα, and IFNβ (Supplementary Table 1). All results were normalized to glyceraldehyde-3-phosphate dehydrogenase (GAPDH) levels and the fold-change for each was determined using the 2^−ΔΔCt^ method comparing to HTNV uninfected mice naive controls (*n* = 5).

### Flow cytometry analysis of cytokines

Spleen cells were prepared, suspended, and maintained in Roswell Park Memorial Institute (RPMI) 1640 (Corning, Corning, NY, USA) supplemented with 10% FBS. Cells were stimulated with 15-mer peptides containing an overlap of 8 amino acid residues that covered the full-length HTNV GP for 8 h, along with a protein transport inhibitor cocktail (added 3 h later)^11,48^. After stimulation, 1 μL of FVD-780 was added, and the cells were incubated for 30 min at 2–8 °C. They were then washed and stained with fluorophore-conjugated antibodies against the surface markers CD3 Monoclonal Antibody (17A2) (eFluor 506, 2.5 μL/100 μL, eBioseience, 69-0032-82), CD4 Monoclonal Antibody (GK1.5) (FITC, 1.25 μL/100 μL, eBioseience, 11-0041-82), and CD8a Monoclonal Antibody (53-6.7) (PE-Cyanine7, 2.5 μL/100 μL, eBioseience, 25-0081-82) for 60 min at room temperature. The cells were then treated with Intracellular Fixation & Permeabilization Buffer (eBioscience) for 30 min, centrifuged at 1000 × *g* for 3 min, and re-suspended. Further, fluorophore conjugated-antibodies against intracellular TNF alpha Monoclonal Antibody (MP6-XT22) (PerCP-eFluor™ 710, 2.5 μL/100 μL, eBioseience, 46-7321-82), IL-4 Monoclonal Antibody (11B11) (PE, 0.625 μL/100 μL, eBioseience, 12-7041-82), and IFN gamma Monoclonal Antibody (XMG1.2) (APC, 0.625 μL/100 μL, eBioseience, 17-7311-82) were added, and incubated for 30 min. After washing, spleen cells were analyzed using a flow cytometer (Agilent).

### Enzyme-linked immunospot assay of cytokines

The cytokines IL-2, IL-4, IL-10 and IFN-γ were determined using ELISpot kits (MABTECH, NackaStrand, Sweden) following the manufacturer’s instructions. The mice were euthanized and their spleens were dissected and ground into a monocyte suspension. The splenocytes were washed and re-suspended following erythrocyte lysis. After blocking the ELISpot plates for 30 min with RPMI-1640 containing 10% FBS at room temperature, 1 × 10^6^ cells/well were transferred to the ELISpot plates and stimulated with 10 μg/mL HTNV Gn/Gc peptide pool for 24 h at 37 °C. Concanavalin A (1 μg/mL) was added as a positive control. Unstimulated cells were used as negative controls. After 24 h of incubation, streptavidin-HRP and fresh substrates were added to the plates. The reaction was stopped by rinsing the plate with deionized water. After drying, the number of spots was determined using an ImmunoSpot® analyzer (Cellular Technology Ltd., Santa Rosa, California, USA).

### Histopathology

For H&E histopathology evaluation, mice were euthanized and their livers and lungs were rapidly isolated and fixed in 4% PFA at 4 °C overnight, followed by paraffin embedding. Sections were stained with H&E to analyze the histopathological changes. Images were captured using a Panoramic MIDI instrument (3D HISTECH, Hungary) and analyzed using SlideViewer V2.6.0 software.

### Statistical analysis

Statistical significance was assigned at *P*-values less than 0.05 using Prism Version 8 (GraphPad Software). For the correlation analysis, Spearman’s correlation coefficients (R^2^) and *P*-values are indicated. Analysis of specific antibody and neutralization titers in mice after vaccination was performed using one-way Analysis of Variance (ANOVA) with Dunnett’s post-hoc test. Differences in viral loads or cytokine levels after HTNV challenge in immunized mice were determined using the Kruskal–Wallis test with Dunn’s post-hoc test.

### Reporting summary

Further information on research design is available in the Nature Research Reporting Summary linked to this article.

### Supplementary information

SUPPLEMENTARY DATA

REPORTING SUMMARY

## Data Availability

All data that support the findings of this study are available from the corresponding author upon reasonable request.
