# Supplementary material for: Single dose recombinant VSV based vaccine elicits robust and durable neutralizing antibody against Hantaan virus
Source: NPJ Vaccines. 2024 Feb 10;9:28. doi: 10.1038/s41541-024-00814-2 (PMC10858903; doi:10.1038/s41541-024-00814-2)
Supplement: Supplementary file 1 — SUPPLEMENTARY DATA [file 41541_2024_814_MOESM1_ESM.pdf]

Single dose recombinant VSV based vaccine elicits robust and durable neutralizing antibody against Hantaan virus

Hui Zhang[1,†], He Liu[1,†], Jing Wei[1,2,†], Yamei Dang[1,†], Yuan Wang[1], Qiqi Yang[1], Liang Zhang[1], Chuantao Ye[1], Bin Wang[3], Xiaolei Jin[4], Linfeng Cheng[1], Hongwei Ma[1], Yangchao Dong[1], Yinghui Li[1], Yinlan Bai[1], Xin Lv[1], Yingfeng Lei[1], Zhikai Xu[1,*], Wei Ye[1,*], Fanglin Zhang[1,*]

[1] Department of Microbiology, School of Preclinical Medicine, Airforce Medical University: Fourth Military Medical University, Xi' an, Shaanxi, China.

[2] Center for Disease Control and Prevention of Shaanxi Province, Xi'an, Shaanxi, China.

[3] Center of Clinical Aerospace Medicine, Airforce Medical University: Fourth Military Medical University, Xi' an, Shaanxi, China.

[4] Student Brigade, School of Preclinical Medicine, Airforce Medical University: Fourth Military Medical University, Xi' an, Shaanxi, China.

[†] These authors contributed equally.

* To whom correspondence should be addressed. Fanglin Zhang, Email: flzhang@fmmu.edu.cn. Correspondence may also be addressed to Wei Ye, Email: virologyyw@fmmu.edu.cn; Zhikai Xu, Email: zhikaixu@fmmu.edu.cn.

SUPPLEMENTARY DATA

Supplementary Table 1. The primer of inflammatory cytokine for RT-qPCR.

Supplementary Figure 1. HFRS convalescent Serum Neutralization of HTNV and rVSV-HTNV-GP

(a–b) Representative neutralization curves of antisera from patients with convalescent HFRS exhibiting inhibitory activity against HTNV (a) or rVSV-HTNV-GP (b) (n = 5 for each panel). Error bars represent the standard error of the mean.

Related to Figure 2.

Supplementary Figure 2. Gating strategy to quantify HTNV GP specific $CD4^+$ T cells in mice splenocytes.

Related to Supplementary Figure 3.

Supplementary Figure 3. HTNV specific T cell immune responses in mice immunized with rVSV-HTNV-GP

(a) Representative flow cytometry analysis results of intracellular IFNγ, TNFα, and IL-4 expressing CD4+ T cell in splenocytes response to HTNV-GP peptides.

(b) Statistical analysis of flow cytometry results. (Data are presented as the mean ± SEMs, n = 3 mice per group; one-way ANOVA with Dunnett’s post-test).

Related to Figure 2.

Supplementary Figure 4. Enzyme-linked immunospot assay of cytokines

(a-d) Secretion of IL-2 (a), IL-4 (b), IL-10 (c) and IFNγ (d) in splenocytes was determined through ELISpot assays under the stimulation of 10 μg/mL HTNV-GP peptide. (Data are presented as the mean ± SEMs, n = 3 mice per group; one-way ANOVA with Dunnett’s post-test).

Related to Figure 2.

Supplementary Figure 5. rVSV-HTNV-GP vaccination reduces viral loads in multiple tissues and protects from HTNV-Induced Inflammation

(a–c) Four weeks after rVSV or rVSV-HTNV-GP immunization or 3 weeks after the last boost of inactivated vaccine, mice were challenged with 1×10^6 FFUs of HTNV via intramuscular administration. Three days later, the tissues were harvested and cytokine IL-6 levels in mice lung (a), kidney (b), and liver (c) tissues were evaluated by RT-qPCR assay.

(d–f) Evaluation of cytokine IL-10 in mice lung (d), kidney (e), and liver (f) tissues by RT-qPCR assay.

(g–i) Viral loads (g), TNFα (h), and IL-1β (i) in mice lung tissue evaluated by RT-qPCR assay.

(j–l) Evaluation of IFNβ (j), IL-6 (k), and IL-10 (l) in mice lung tissue by RT-qPCR assay. Data are shown as fold change in gene expression compared to fully naive, age-matched animals after normalization to GAPDH. (n = 5 mice per group; data of viral loads are represented as the means ± SEMs, and data of cytokine are represented as median values. Kruskal–Wallis test with Dunn's post-test: *P<0.05, **P<0.01, ***P<0.001).

Related to Figure 4.

Supplementary Figure 6. rVSV-HTNV-GP provides long-lasting protection to mice

(a–c) Evaluation of cytokine TNFα in mice lung (a), kidney (b), and liver (c) tissues by RT-qPCR assay.

(d–f) Evaluation of cytokine IL-1β in mice lung (d), kidney (e), and liver (f) tissues by RT-qPCR assay.

(g–i) Evaluation of cytokine IL-6 in mice lung (g), kidney (h), and liver (i) tissues by RT-qPCR assay.

(j–l) Evaluation of cytokine IL-10 in mice lung (j), kidney (k), and liver (l) tissues by RT-qPCR assay.

Data are shown as fold-changes in gene expression compared to fully naïve, age-matched animals after normalization to GAPDH. (n = 5 mice per group; data are represented as median values; Kruskal–Wallis test with Dunn's post-hoc test: *P<0.05, **P<0.01).

Related to Figure 8.

Supplementary Figure 7. The full and un-cropped images of Fig. 1f and Fig. 1g

Supplementary Table 1. The primer of inflammatory cytokine for RT-qPCR.

Primer	Sequences
IFNβ /forward	ATGAGTGGTGGTTGCAGGC
IFNβ /reverse	TGACCTTTCAAATGCAGTAGATTCA
IL-6 /forward	TGCAAGAGACTTCCATCCAGTTG
IL-6 /reverse	TAAGCCTCCGACTTGTGAAGTGGT
IL-1β /forward	TCATTGTGGCTGTGGAGAAGC
IL-1β /reverse	AATGGGAACGTCACACACCAG
IL-10 /forward	TGAATTCCCTGGGTGAGAAGCTGA
IL-10 /reverse	TGGCCTTGTAGACACCTTGGTCTT
TNFα /forward	GCCTCTTCTCATTCCTGCTT
TNFα /reverse	CTCCTCCACTTGGTGGTTTG
GAPDH /forward	GGTGAAGGTCGGTGTCAACG
GAPDH /reverse	CAAAGTTGTCATGGATGACC

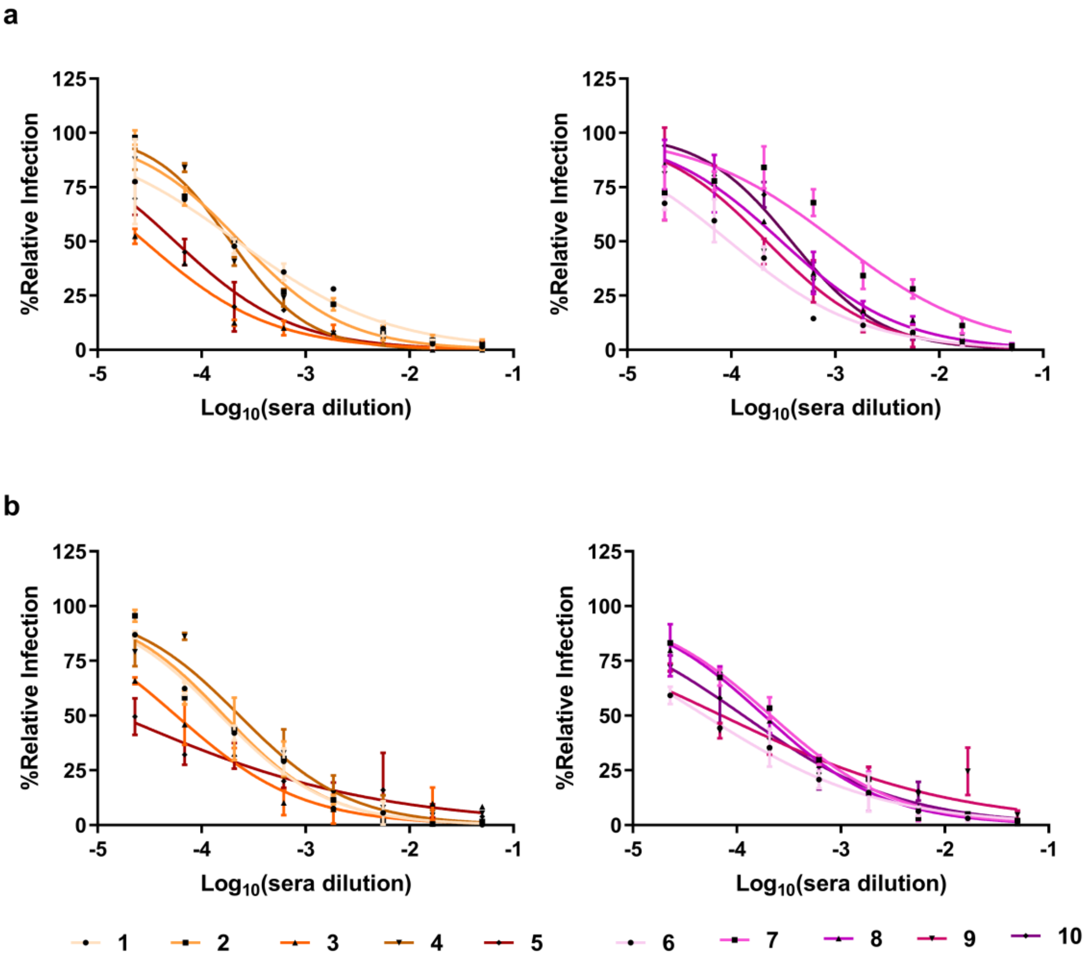

Supplementary Figure 1. HFRS convalescent Serum Neutralization of HTNV and rVSV-HTNV-GP

(a–b) Representative neutralization curves of antisera from patients with convalescent HFRS exhibiting inhibitory activity against HTNV (a) or rVSV-HTNV-GP (b) (n = 5 for each panel). Error bars represent the standard error of the mean.

Related to Figure 2.

Gating strategy

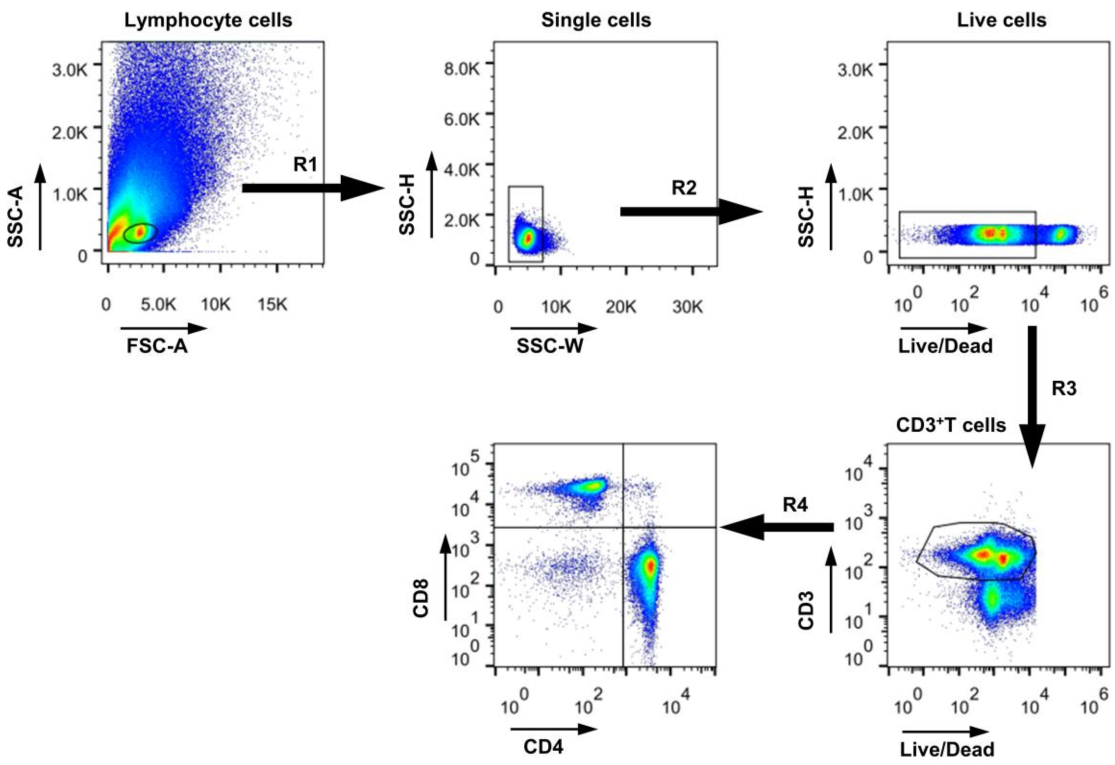

Supplementary Figure 2. Gating strategy to quantify HTNV GP specific CD4⁺ T cells in mice splenocytes.

Related to Supplementary Figure 3.

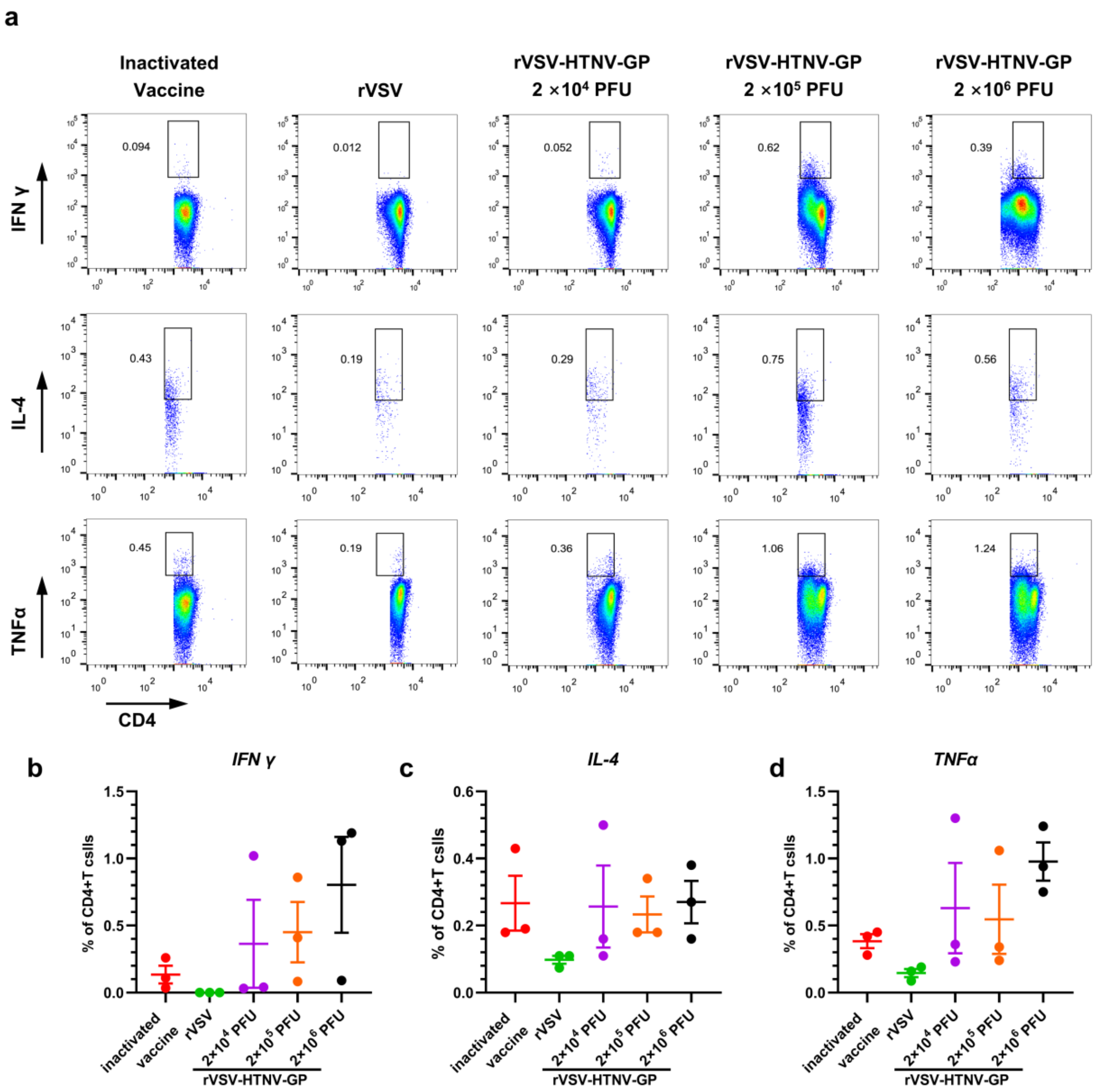

Supplementary Figure 3. HTNV specific T cell immune responses in mice immunized with rVSV-HTNV-GP

(a) Representative flow cytometry analysis results of intracellular IFNγ, TNFα, and IL-4 expressing CD4+ T cell in splenocytes response to HTNV-GP peptides.

(b) Statistical analysis of flow cytometry results. (Data are presented as the mean ± SEMs, n = 3 mice per group; one-way ANOVA with Dunnett's post-test).

Related to Figure 2.

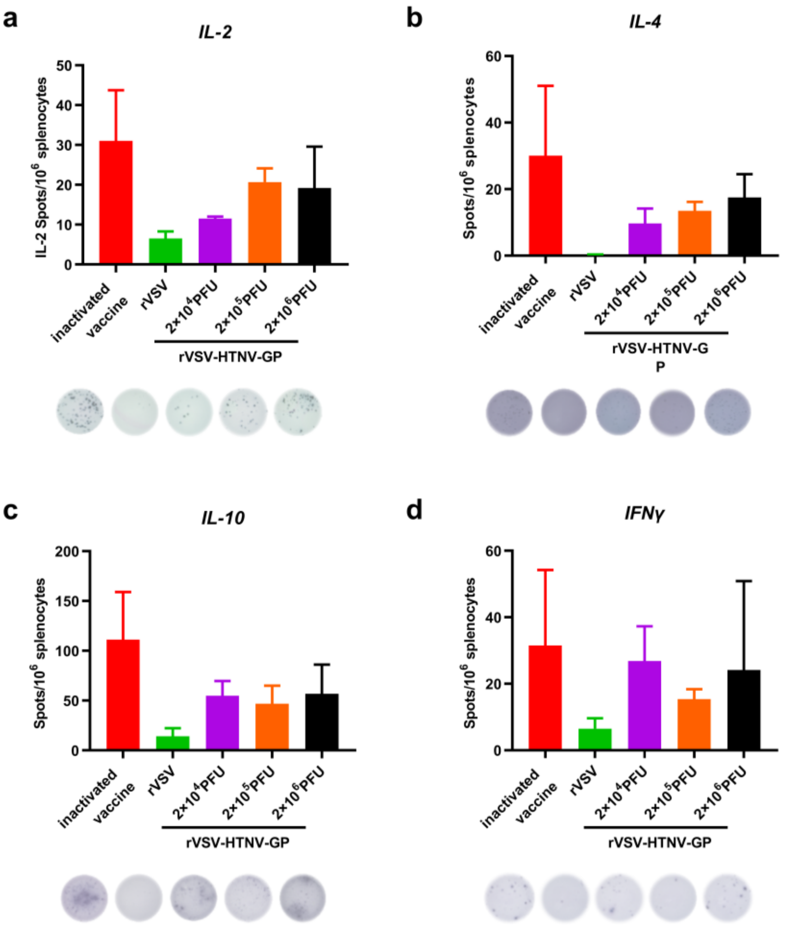

Supplementary Figure 4. Enzyme-linked immunospot assay of cytokines

(a-d) Secretion of IL-2 (a), IL-4 (b), IL-10 (c) and IFNγ (d) in splenocytes was determined through ELISpot assays under the stimulation of 10 μg/mL HTNV-GP peptide. (Data are presented as the mean ± SEMs, n = 3 mice per group; one-way ANOVA with Dunnett's post-test).

Related to Figure 2.

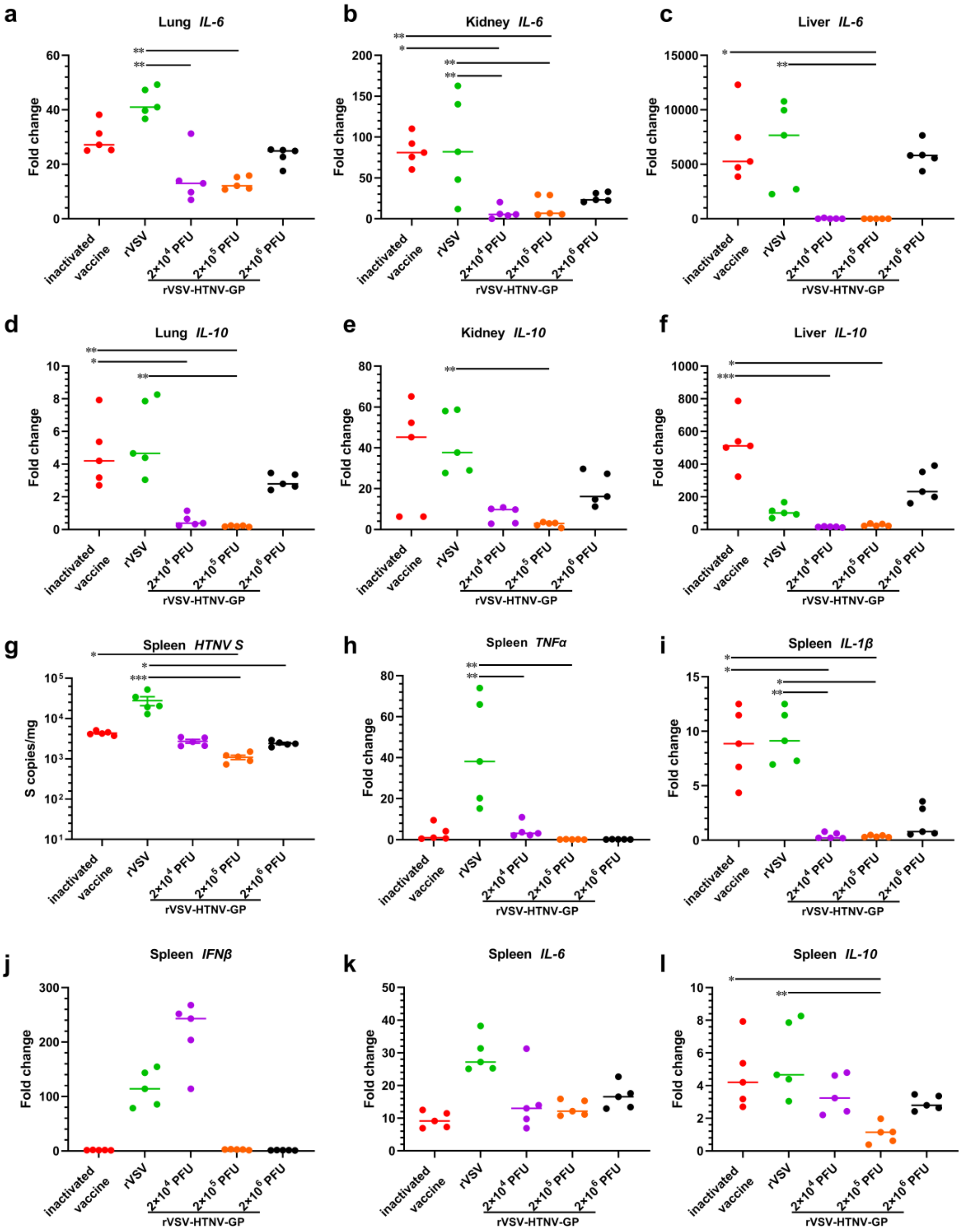

Supplementary Figure 5. rVSV-HTNV-GP vaccination reduces viral loads in multiple tissues and protects from HTNV-Induced Inflammation

(a–c) Four weeks after rVSV or rVSV-HTNV-GP immunization or 3 weeks after the last boost of inactivated vaccine, mice were challenged with $1×10^6$ FFUs of HTNV via intramuscular administration.

Three days later, the tissues were harvested and cytokine IL-6 levels in mice lung (a), kidney (b), and liver (c) tissues were evaluated by RT-qPCR assay.

(d–f) Evaluation of cytokine IL-10 in mice lung (d), kidney (e), and liver (f) tissues by RT-qPCR assay.

(g–i) Viral loads (g), TNFα (h), and IL-1β (i) in mice lung tissue evaluated by RT-qPCR assay.

(j–l) Evaluation of IFNβ (j), IL-6 (k), and IL-10 (l) in mice lung tissue by RT-qPCR assay.

Data are shown as fold change in gene expression compared to fully naive, age-matched animals after normalization to GAPDH. ($n = 5$ mice per group; data of viral loads are represented as the means ± SEMs, and data of cytokine are represented as median values. Kruskal–Wallis test with Dunn’s post-test: *$P<0.05$, **$P<0.01$, ***$P<0.001$).

Related to Figure 4.

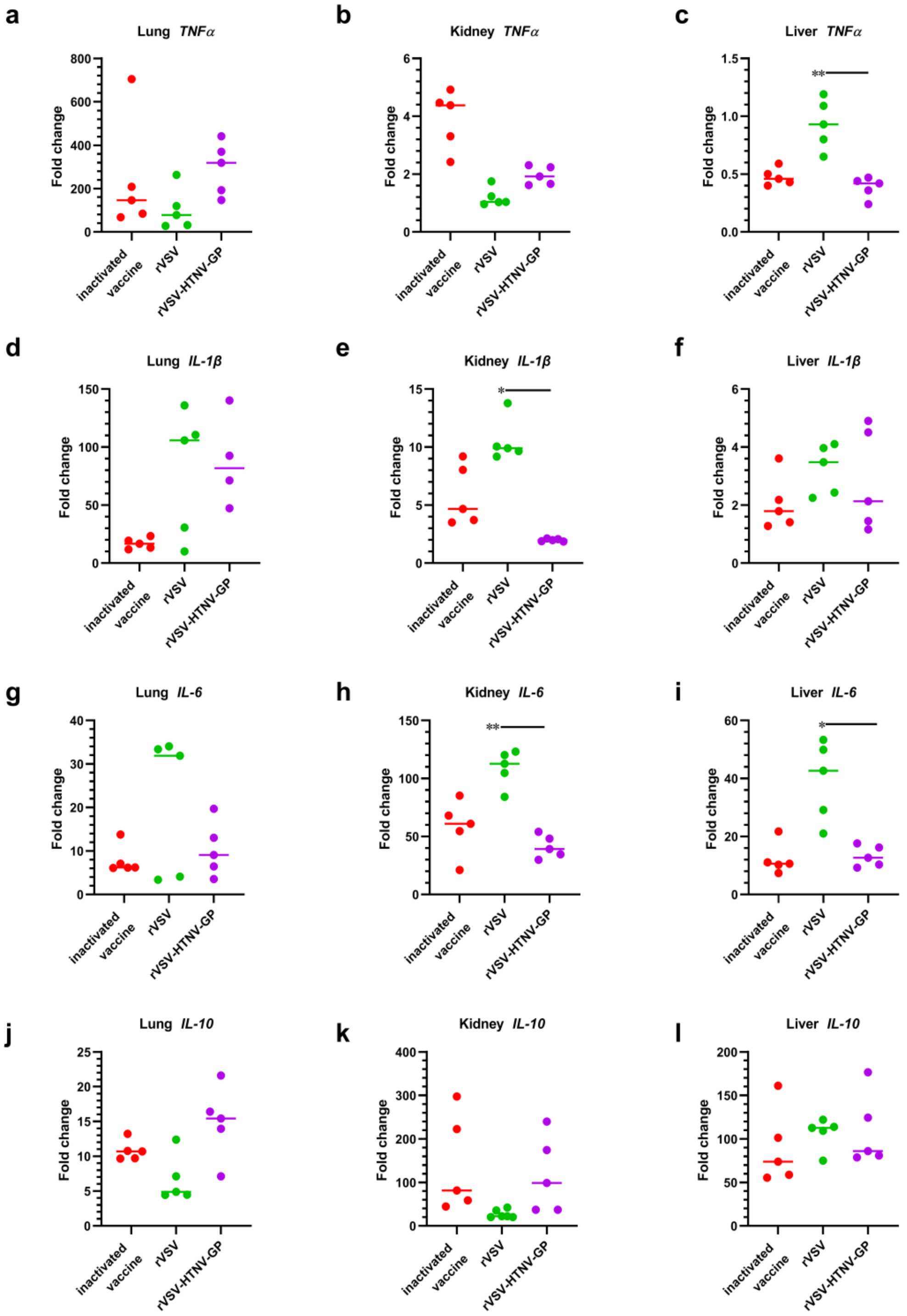

Supplementary Figure 6. rVSV-HTNV-GP provides long-lasting protection to mice

(a–c) Evaluation of cytokine TNFα in mice lung (a), kidney (b), and liver (c) tissues by RT-qPCR assay.

(d–f) Evaluation of cytokine IL-1β in mice lung (d), kidney (e), and liver (f) tissues by RT-qPCR assay.

(g–i) Evaluation of cytokine IL-6 in mice lung (g), kidney (h), and liver (i) tissues by RT-qPCR assay.

(j–l) Evaluation of cytokine IL-10 in mice lung (j), kidney (k), and liver (l) tissues by RT-qPCR assay.

Data are shown as fold-changes in gene expression compared to fully naïve, age-matched animals after normalization to GAPDH. (n = 5 mice per group; data are represented as median values; Kruskal–Wallis test with Dunn’s post-hoc test: *P<0.05, **P<0.01).

Related to Figure 8.

Figure 1 f

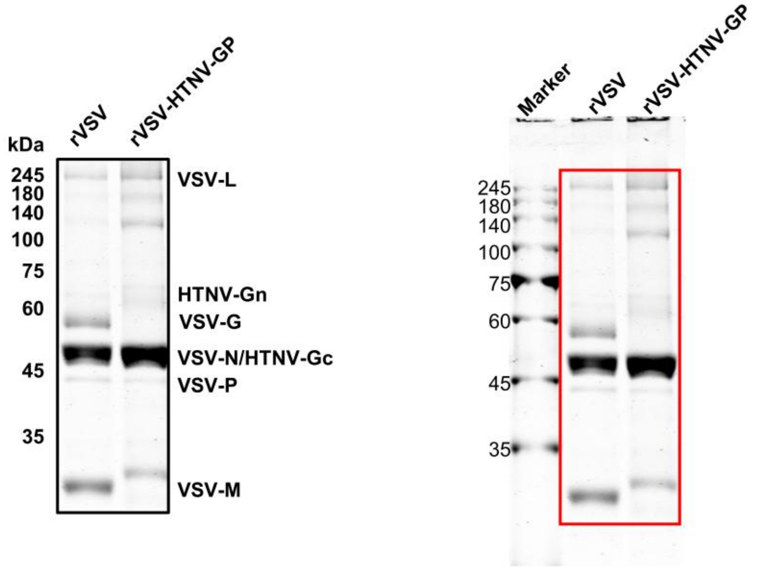

Figure 1 g

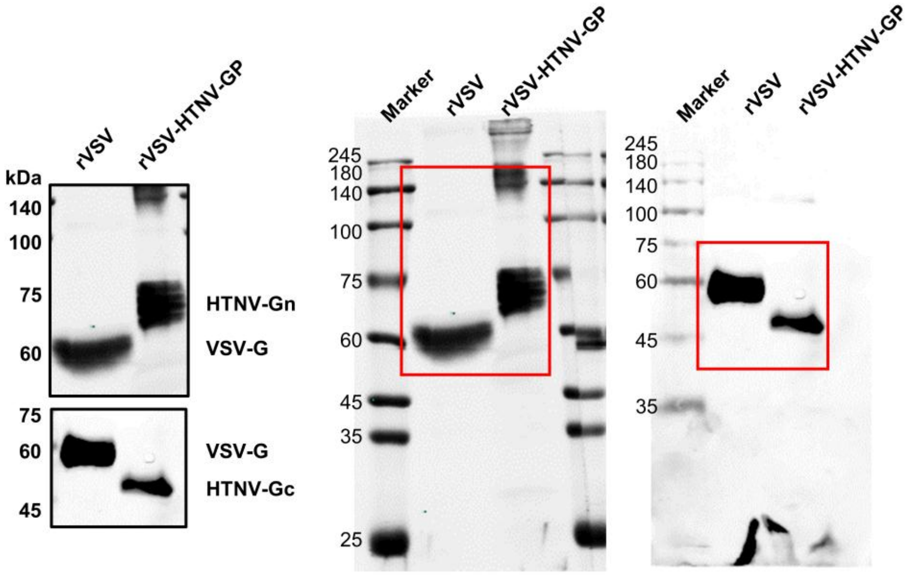

Supplementary Figure 7. The full and un-cropped images of Figure.1F and Figure.1G
