# Supplementary material for: Single dose recombinant VSV based vaccine elicits robust and durable neutralizing antibody against Hantaan virus
Source: NPJ Vaccines. 2024 Feb 10;9:28. doi: 10.1038/s41541-024-00814-2 (PMC10858903; doi:10.1038/s41541-024-00814-2)
Supplement: Supplementary file 2 — REPORTING SUMMARY [file 41541_2024_814_MOESM2_ESM.pdf]

nature portfolio

NPJVACCINES-03385
Corresponding author(s): Zhikai Xu
Wei Ye
Fanglin Zhang

Last updated by author(s): Nov 16, 2023

Reporting Summary

Nature Portfolio wishes to improve the reproducibility of the work that we publish. This form provides structure for consistency and transparency in reporting. For further information on Nature Portfolio policies, see our Editorial Policies and the Editorial Policy Checklist.

Statistics

For all statistical analyses, confirm that the following items are present in the figure legend, table legend, main text, or Methods section.

n/a	Confirmed	
☐	☒	The exact sample size (n) for each experimental group/condition, given as a discrete number and unit of measurement
☐	☒	A statement on whether measurements were taken from distinct samples or whether the same sample was measured repeatedly
☐	☒	The statistical test(s) used AND whether they are one- or two-sided<br>*Only common tests should be described solely by name; describe more complex techniques in the Methods section.*
☒	☐	A description of all covariates tested
☒	☐	A description of any assumptions or corrections, such as tests of normality and adjustment for multiple comparisons
☐	☒	A full description of the statistical parameters including central tendency (e.g. means) or other basic estimates (e.g. regression coefficient) AND variation (e.g. standard deviation) or associated estimates of uncertainty (e.g. confidence intervals)
☒	☐	For null hypothesis testing, the test statistic (e.g. F, t, r) with confidence intervals, effect sizes, degrees of freedom and P value noted<br>*Give P values as exact values whenever suitable.*
☒	☐	For Bayesian analysis, information on the choice of priors and Markov chain Monte Carlo settings
☒	☐	For hierarchical and complex designs, identification of the appropriate level for tests and full reporting of outcomes
☒	☐	Estimates of effect sizes (e.g. Cohen's d, Pearson's r), indicating how they were calculated

Our web collection on statistics for biologists contains articles on many of the points above.

Software and code

Policy information about availability of computer code

Data collection	no software was used
Data analysis	SlideViewer V2.6.0<br>FlowJo v10<br>Graphpad Prism 8.0<br>Microsoft Office 2016

For manuscripts utilizing custom algorithms or software that are central to the research but not yet described in published literature, software must be made available to editors and reviewers. We strongly encourage code deposition in a community repository (e.g. GitHub). See the Nature Portfolio guidelines for submitting code & software for further information.

Data

Policy information about availability of data

All manuscripts must include a data availability statement. This statement should provide the following information, where applicable:

- Accession codes, unique identifiers, or web links for publicly available datasets
- A description of any restrictions on data availability
- For clinical datasets or third party data, please ensure that the statement adheres to our policy

All data that support the findings of this study are available from the corresponding author upon reasonable request.

Research involving human participants, their data, or biological material

Policy information about studies with human participants or human data. See also policy information about sex, gender (identity/presentation), and sexual orientation and race, ethnicity and racism.

Reporting on sex and gender	
Reporting on race, ethnicity, or other socially relevant groupings	
Population characteristics	
Recruitment	
Ethics oversight	

Note that full information on the approval of the study protocol must also be provided in the manuscript.

Field-specific reporting

Please select the one below that is the best fit for your research. If you are not sure, read the appropriate sections before making your selection.

☒ Life sciences ☐ Behavioural & social sciences ☐ Ecological, evolutionary & environmental sciences

For a reference copy of the document with all sections, see nature.com/documents/nr-reporting-summary-flat.pdf

Life sciences study design

All studies must disclose on these points even when the disclosure is negative.

Sample size	The sample size of animal studies was chosen taking into account previous works in this area.<br>J Virol. 2011 Dec;85(23):12781-91. doi: 10.1128/JVI.00794-11.<br>Vaccine. 2015 Jun 26;33(29):3367-76. doi: 10.1016/j.vaccine.2015.05.007.<br>Antiviral Res. 2018 Feb:150:174-182. doi: 10.1016/j.antiviral.2017.12.011.<br>Front Cell Infect Microbiol. 2020 Jul 8:10:333. doi: 10.3389/fcimb.2020.00333.
Data exclusions	No data have been excluded from the analyses
Replication	All the analyses have been replicated
Randomization	Animals were allocated in experimental groups randomly
Blinding	The researchers were not blinded to the study.

Behavioural & social sciences study design

All studies must disclose on these points even when the disclosure is negative.

Study description	The serum samples were used for antigenic comparison between rVSV-HTNV-GP and HTNV
Research sample	Serum collected from convalescence HFRS patients
Sampling strategy	N/A
Data collection	N/A
Timing	convalescence
Data exclusions	No data have been excluded from the analyses
Non-participation	N/A
Randomization	N/A

Ecological, evolutionary & environmental sciences study design

All studies must disclose on these points even when the disclosure is negative.

Study description	N/A
Research sample	N/A
Sampling strategy	N/A
Data collection	N/A
Timing and spatial scale	N/A
Data exclusions	N/A
Reproducibility	N/A
Randomization	N/A
Blinding	N/A

Did the study involve field work? ☐ Yes ☒ No

Field work, collection and transport

Field conditions	
Location	
Access & import/export	
Disturbance	

Reporting for specific materials, systems and methods

We require information from authors about some types of materials, experimental systems and methods used in many studies. Here, indicate whether each material, system or method listed is relevant to your study. If you are not sure if a list item applies to your research, read the appropriate section before selecting a response.

Materials & experimental systems

n/a	Involved in the study	
☐	☒	Antibodies
☐	☒	Eukaryotic cell lines
☒	☐	Palaeontology and archaeology
☐	☒	Animals and other organisms
☒	☐	Clinical data
☒	☐	Dual use research of concern
☒	☐	Plants

Methods

n/a	Involved in the study	
☒	☐	ChIP-seq
☐	☒	Flow cytometry
☒	☐	MRI-based neuroimaging

Antibodies

Antibodies used

anti-VSV-G tag: EPR28362-204 ,abcam,Cat number:309106
IRDy 680RD goat anti-mouse secondary antibodies, LI-COR, Cat number: 926-68070
IRDye 800CW Goat anti-rabbit secondaries, LI-COR, Cat number: 926-32211
Cy3-conjugated goat anti-rabbit Ig G, Sangon Biotech, Cat number: D111018
Cy3-conjugated goat anti-mouse Ig G, Sangon Biotech, Cat number: D111024
FITC-conjugated goat anti-mouse Ig G, Sangon Biotech, Cat number: D110105
HRP-conjugated goat anti-mouse Ig G, Sangon Biotech, Cat number: D110087
Hoechst 33258, YEASEN, Cat number: 40729ES10

	Anti-mose CD3 Monoclonal Antibody: 17A2 clone, eBioscience, Cat number: 69-0032-82<br>Anti-mouse CD4 Monoclonal Antibody: GK1.5 clone, eBIOscience, Cat number: 11-0041-82<br>Anti-mouse IL-4 Monoclonal Antibody: 11B11 clone, eBioscience, Cat number:17-7041-82<br>Anti-mouse TNFa Monoclonal Antibody:MP6-XT22 clone, eBIOscience, Cat number: 17-7321-82<br>Anti-mouse IFN gama Monoclonai Antibody: XMG1.2 clone, eBioscience, Cat number: 17-7311-82<br>Anti-mouse CD8a Monoclonal Antibody: 53-6.7, eBioscience, Cat number: 11-0081-82
Validation	We follow manufactures instruction to use the above listed antibodies. All antibodies work well.

Eukaryotic cell lines

Policy information about cell lines and Sex and Gender in Research

Cell line source(s)	BHK-21 cells (ATCC) were used in the in vitro transfectiom assay;<br>Vero E6 cells (ATCC) were used in neutralization assay;<br>A549 cells (ATCC), Huh7 cells (Procell), HeLa cells (ATCC)<br>and HUVEC (ScienCell) were used in the in vitro infection assay.
Authentication	Authentication by vendors.
Mycoplasma contamination	Cell lines provided by ATCC, SciencCell or Procell are guaranteed to by mycoplasma negative.<br>We used standard antobiotics treatment in media as recommended by the vendors.
Commonly misidentified lines<br>(See ICLAC register)	NA

Palaeontology and Archaeology

Specimen provenance	
Specimen deposition	
Dating methods	

☐ Tick this box to confirm that the raw and calibrated dates are available in the paper or in Supplementary Information.

Ethics oversight	

Note that full information on the approval of the study protocol must also be provided in the manuscript.

Animals and other research organisms

Policy information about studies involving animals; ARRIVE guidelines recommended for reporting animal research, and Sex and Gender in Research

Laboratory animals	Female BALB/c mice, aged 6-8 weeks, used in this shudy were purchased from the<br>Laboraty Animal Centre of Air Force Medical University.
Wild animals	The study did not involve wild animals.
Reporting on sex	BALB/c mice used in this study were all female.
Field-collected samples	The study did not involve field-collected samples.
Ethics oversight	All animal studies were carried out in strict accordance with the recommendation in the Guide for the Care<br>and Use of Laboratory Animals of the Ministry of Science and Technology of the Peoples Republic of China.<br>The protocols for animal studies were approved by the Committee on the Ethics of Laboratory Animal<br>Centre of Air Force Medical University (Approval number: 20200403).

Note that full information on the approval of the study protocol must also be provided in the manuscript.

Clinical data

Policy information about clinical studies
All manuscripts should comply with the ICMJE guidelines for publication of clinical research and a completed CONSORT checklist must be included with all submissions.

Clinical trial registration	
Study protocol	

Data collection

Outcomes

Dual use research of concern

Policy information about dual use research of concern

Hazards

Could the accidental, deliberate or reckless misuse of agents or technologies generated in the work, or the application of information presented in the manuscript, pose a threat to:

No	Yes	
☒	☐	Public health
☒	☐	National security
☒	☐	Crops and/or livestock
☒	☐	Ecosystems
☒	☐	Any other significant area

Experiments of concern

Does the work involve any of these experiments of concern:

No	Yes	
☒	☐	Demonstrate how to render a vaccine ineffective
☒	☐	Confer resistance to therapeutically useful antibiotics or antiviral agents
☒	☐	Enhance the virulence of a pathogen or render a nonpathogen virulent
☒	☐	Increase transmissibility of a pathogen
☒	☐	Alter the host range of a pathogen
☒	☐	Enable evasion of diagnostic/detection modalities
☒	☐	Enable the weaponization of a biological agent or toxin
☒	☐	Any other potentially harmful combination of experiments and agents

Plants

Seed stocks

Novel plant genotypes

Authentication

ChIP-seq

Data deposition

☐ Confirm that both raw and final processed data have been deposited in a public database such as GEO.

☐ Confirm that you have deposited or provided access to graph files (e.g. BED files) for the called peaks.

Data access links
May remain private before publication.

Files in database submission

Genome browser session
(e.g. UCSC)

Methodology

Replicates	
Sequencing depth	
Antibodies	
Peak calling parameters	
Data quality	
Software	

Flow Cytometry

Plots

Confirm that:

☒ The axis labels state the marker and fluorochrome used (e.g. CD4-FITC).

☒ The axis scales are clearly visible. Include numbers along axes only for bottom left plot of group (a 'group' is an analysis of identical markers).

☒ All plots are contour plots with outliers or pseudocolor plots.

☒ A numerical value for number of cells or percentage (with statistics) is provided.

Methodology

Sample preparation	The BALB/c mice were randomly divided into five groups (n=5 per group). Mice were intraperitoneally (i.p.) injected with the HFRS-inactivated vaccine, rVSV, or rVSV-HTNV-GP, and 200 uL ()100ug HFRS inactivated vaccine was administred three times at 3 week intervals. Then, 2x104 PFUs of rVSV were administred. For rVSV-HTNV-GP, three different doses of 2x104, 2x105, or 2x106 PFUs were adiministred. 4 weeks after rVSV or rVSV-HTNV-GP administration, and 3 weeks after the vaccination regime was completed, the animals were euthanized, spleen cells were collected, and cytokines in CD4+T cells were evaluated using flow cytometry.<br>Spleen cells were prepared, suspened, and maintained in RPMI 1640 supplemented with 10% FBS. Cells were stimulated with 15-mer peptides containing an overlap of amino acid residues than covered the full-length HTNV GP for 8h, along with a protein transport inhibitor cocktail (added 3 h later). After stimulation,1uL od FVD-780 (Fixable Viability Dye eFluor 780, eBioscience, Cat number: 65-0865-18) was added, and the cells were incubated for 30 min, They were then washed and stained with antibodies against the surface markers CD3 Monoclonal antibody (17A2), eFluor 506, CD4 Monoclonal Antibody (GK1.5),FITC, CD8a M[illegible]onoclonal Antibody (53-6.7), PE-Cyanine 7 for 60 min at room temperature. The cells were then treated with Intracellular Fixation& Permeabilization Buffer (eBioscience) for 30 min, centrifuged at 1,000xg for 3 min, and re-suspened. further, IL-4 Monoclonal Antibody (11811),PE, IFN gama Mnoclonal Antibody (XMG1.2), APC and TNFa Monoclonal Antibody (MP6-XT22), perCP-eFluor 710 were added, and incubated for 30 min. After washing, spleen cells were analyzed using a flow cytometer (Agilent).
Instrument	NovoExpress For NovoCyte/Quanteon/Adcanteon Flow Cytometers (Agilent)
Software	Data were analyzed with FlowJo software V10.
Cell population abundance	N/A
Gating strategy	The gating strategy was referenced by the article. Liang Qu et al, Circular RNA vaccines against SARS-CoV-2 and emerging variants. Cell. 2022 May 12;185(10): 1728-1722.e16. doi: 10.1016/j.cell.2022.03.044.

☒ Tick this box to confirm that a figure exemplifying the gating strategy is provided in the Supplementary Information.

Magnetic resonance imaging

Experimental design

Design type	
Design specifications	
Behavioral performance measures	

Acquisition

Imaging type(s)

Field strength

Sequence & imaging parameters

Area of acquisition

Diffusion MRI ☐ Used ☒ Not used

Preprocessing

Preprocessing software

Normalization

Normalization template

Noise and artifact removal

Volume censoring

Statistical modeling & inference

Model type and settings

Effect(s) tested

Specify type of analysis: ☐ Whole brain ☐ ROI-based ☐ Both

Statistic type for inference

(See Eklund et al. 2016)

Correction

Models & analysis

n/a	Involved in the study
☒	☐ Functional and/or effective connectivity
☒	☐ Graph analysis
☒	☐ Multivariate modeling or predictive analysis

Functional and/or effective connectivity

Graph analysis

Multivariate modeling and predictive analysis
